# Reliability of reporting differences in degenerative MRI findings of the lumbar spine from the supine to the upright position

**DOI:** 10.1007/s00256-022-04060-2

**Published:** 2022-05-10

**Authors:** Klaus Doktor, Jan Hartvigsen, Mark Hancock, Henrik Wulff Christensen, Ulrich Fredberg, Eleanor Boyle, Morten Kindt, Lau Brix, Tue Secher Jensen

**Affiliations:** 1 Department of Sport Sciences and Clinical Biomechanics, Research Unit of Clinical Biomechanics, University of Southern Denmark, Odense, Denmark; 2 Chiropractic Knowledge Hub, University of Southern Denmark, Odense, Denmark; 3 Diagnostic Centre, University Research Clinic for Innovative Patient Pathways, Silkeborg Regional Hospital, Aarhus University, Aarhus, Denmark; 4 Faculty of Medicine and Health Sciences, Macquarie University, Sydney, Australia; 5 The Rheumatology Research Unit, Odense University Hospital, University of Southern Denmark, Odense, Denmark; 6 Department of Procurement and Clinical Engineering, Region Midt, Aarhus, Denmark; 7 Department of Clinical Medicine, Aarhus University, Aarhus, Denmark

**Keywords:** Agreement, Reliability, Reproducibility, Lumbar spine, Upright MRI, Positional MRI

## Abstract

**Objective:**

To determine the inter-rater reliability of identifying differences and types of differences in lumbar degenerative findings comparing supine and upright MRI.

**Materials and methods:**

Fifty-nine participants, low back pain patients (LBP) with or without leg pain and no-LBP individuals were consecutively enrolled to receive supine and upright MRI of the lumbar spine. Three raters independently evaluated the MRIs for degenerative spinal pathologies and compared for differences. Presence/absence of degenerative findings were recorded for all supine and upright images, and then differences from the supine to the upright positions were classified into no-change, appeared, disappeared, worsened, or improved at each individual disc level. Reliability and agreement were calculated using Gwet’s agreement coefficients (AC_1_ or AC_2_) and absolute agreement.

**Results:**

Inter-rater reliability of evaluating differences in eight degenerative lumbar findings comparing the supine and upright MRI position, ranged from 0.929 to 0.996 according to Gwet’s agreement coefficients (AC_2_). The total number of positive MRI findings in the supine position ranged from 270 to 453, with an average of 366 per rater. Observed differences from supine to upright MRI ranged from 18 to 80, with an average of 56 per rater.

**Conclusion:**

Inter-rater reliability was found overall acceptable for classification of differences in eight types of degenerative pathology observed with supine and upright MRI of the lumbar spine. Results were primarily driven by high numbers and high reliability of rating negative findings, whereas agreement regarding positive findings and positive positional differences was lower.

**Supplementary Information:**

The online version contains supplementary material available at 10.1007/s00256-022-04060-2.

## Introduction

It has been suggested that conventional supine MRI may underestimate the presence and degree of gravity-dependent degenerative spinal pathology due to the dynamic nature of some degenerative entities such as disc herniation and scoliosis [1–4]. There is evidence that upright MRI improves the correlation between image findings and patient symptoms beyond supine MRI [3, 5]. However, upright MRI may also be associated with lower sensitivity to serious findings, due to increased motion artifact, and lower image quality [5]. There are no systematic and critical reviews that have evaluated these issues, but three recent narrative reviews have argued for the value of upright MRI [6–8].

Previous studies have investigated the inter-rater reliability of supine MRI findings of the lumbar spine and identified significant variability across degenerative conditions and raters [9, 10]. Hansen et al., [11] assessed supine and upright MRIs on 56 LBP-patients (224 disc levels) with and without sciatica/radiculopathy and found that inter-rater reliability of upright MRI findings of degenerative lumbar spine pathologies was acceptable (kappa > 0.60) for most findings investigated, whereas positional or grading differences in findings from supine to upright position had unacceptable reliability (kappa < 0.60). Since this reliability study was the only publication, we identified comparing supine and upright MRI of the lumbar spine; the objectives of our study was to determine the inter-rater reliability and absolute agreement of lumbar degenerative findings comparing supine and upright MRI.

## Materials and methods

### Study design

This is a fully crossed inter-rater reliability study reported according to the Guidelines for Reporting Reliability and Agreement Studies (GRRAS) [12].

### The study target population

Participants included in this study were a subset of patients with LBP and persons with no LBP living in the Central Denmark Region and enrolled in our main comparative diagnostic test accuracy study. The study had a paired design for index test A (supine MRI) and index test B (upright MRI) in diagnosis of degenerative findings of the lumbar spine. Both studies (reliability and diagnostic accuracy) were carried out at the Department of Radiology, Diagnostic Centre, University Research Clinic for Innovative Patient Pathways, Silkeborg Regional Hospital, Denmark.

The inclusion criteria for LBP patients were (1) referred for MRI from the primary care sector with LBP (with or without back-related leg pain); (2) presence of LBP symptoms for more than 4 weeks; (3) 18 to 60 years of age at the time of consent; (4) not currently waiting for surgery or another advanced hospital procedure indicating specific disease; (5) no suspicion of serious pathology causing symptoms (i.e., cancer, infection or inflammatory arthritis); (6) able to stand for at least 20 min; and (7) able to read and write Danish.

Excluding (1–3) above, the same criteria were used for no-LBP persons with an additional criterion: No presence of LBP for the previous 12 months causing lost workdays. The LBP patients were recruited consecutively from the list of electronic referrals to MRI at the department. No LBP persons were recruited from either the local school of nursing, employees at the hospital, or workplace environments in Silkeborg Municipality/City, Denmark. Most of the recruitment was carried out using posters and by personal communication. All participants provided informed consent via REDCap (Research Electronic Data Capture) on I-pads and completed the electronic questionnaire before imaging procedures could be carried out. I-pads and questions were dealt with by the MRI staff or secretaries.

### Sample selection for the reliability study

**Fig. 1:**
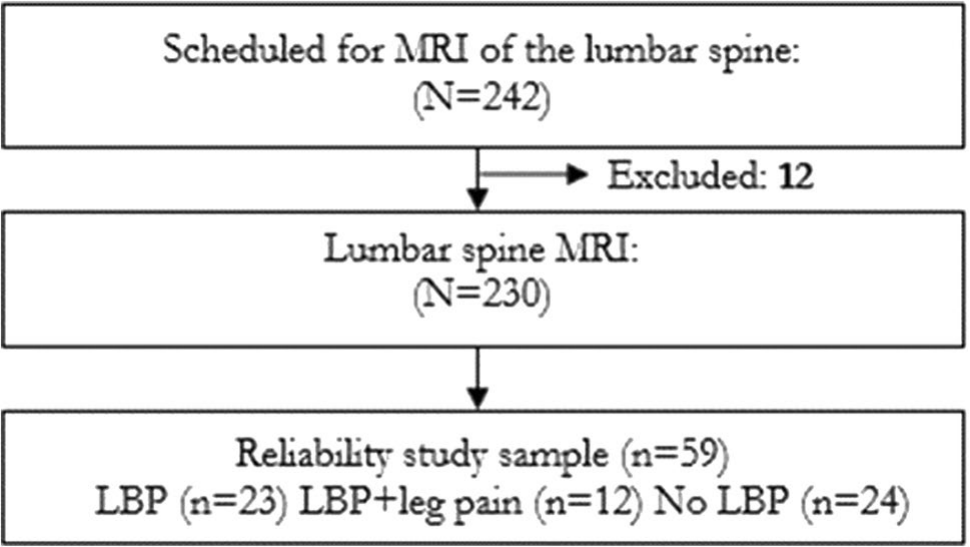
Flowchart of the reliability study sample inclusion for supine and upright MRI

A total of 242 individuals accepted the invitation to participate in the main study. Six participants were excluded because of age over 60 years and another six participants because of technical problems with their baseline questionnaires or inability to complete the MRI procedures. The remaining 230 individuals defined the study population. Of these, the first 59 consecutive participants were included in the inter-rater reliability study from February 26 to April 26, 2018. An overview of the recruitment and exclusion procedure of participants is provided in Fig. 1.

### MRI-procedures

**Fig. 2:**
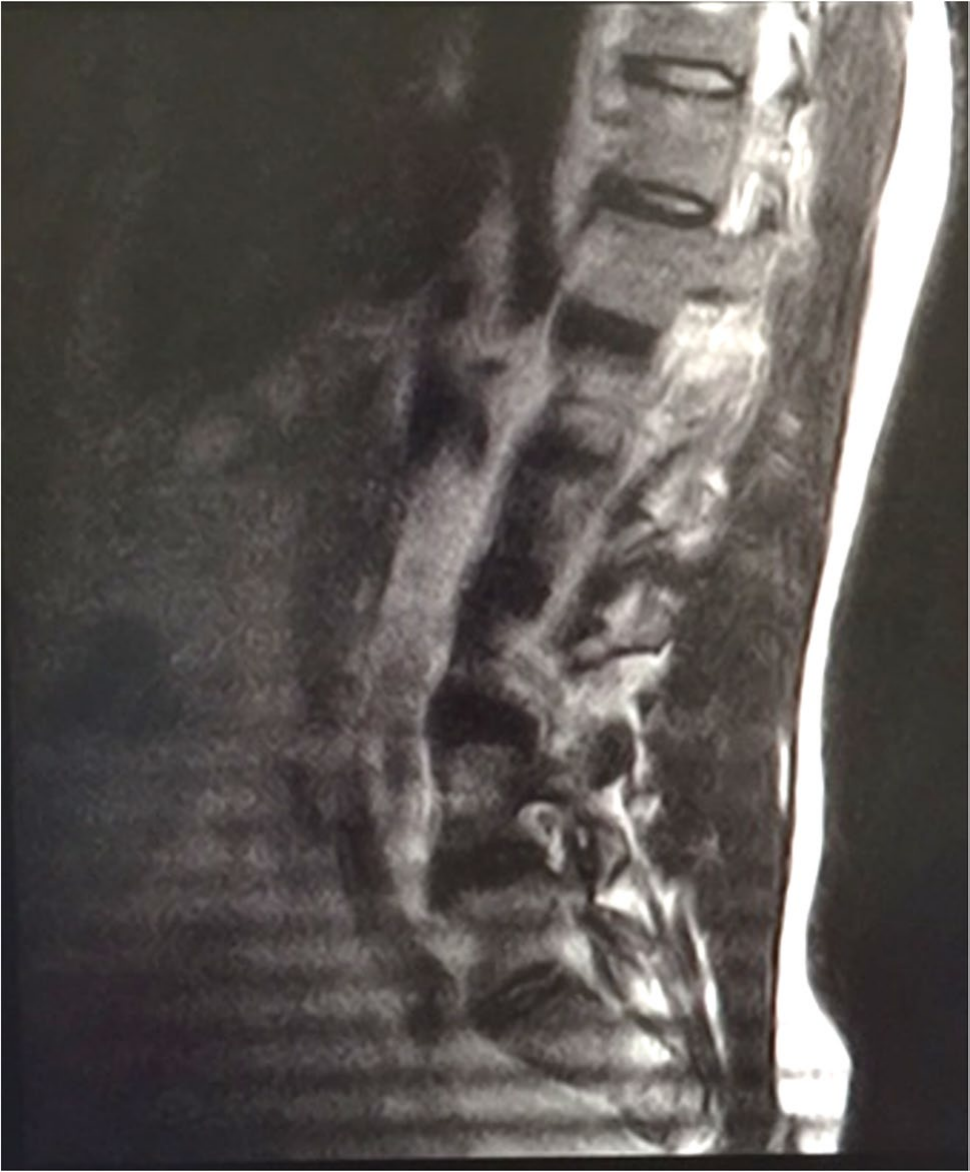
Example of decreased image quality due to episodic technical issues with the 0.5 T upright MRI unit

All participants received MRI in the supine position and the upright position. The LBP patients were scanned in the supine position in either a Siemens Avanto.fit 1.5 T (software release E11c) or a Siemens Skyra 3 T MRI system (Software release E11a, Siemens Healthineers GmbH, Erlangen, Germany) and then in the upright position in an open MRI unit: Paramed MROpen 0.5 T (Paramed Medical Systems, Genoa, Italy). Dedicated spine coils were used for all examinations to ensure optimal image quality. The supine and upright MRI procedures were performed on the same day, but in a few cases, due to technical problems with the OpenMRI unit, the upright procedure was delayed up to 5 days. The no-LBP individuals were scanned supine and upright in the open MRI unit on the same day. The imaging protocols for the two conventional MRI systems (1.5 T and 3.0 T) both included a sagittal 2D T2W Turbo Spin Echo (TSE) sequence as well as an axial 2D T2W TSE sequence. The sagittal sequence on the 3.0 T MRI system included the DIXON fat suppression technique. In addition, a sagittal 2D T1W TSE sequence was added to the 1.5 T protocol, while the 3.0 T protocol also included a sagittal 2D T1W Short TI Inversion Recovery (STIR) sequence. The upright 0.5 T MRI system acquired images using a sagittal 2D T2W Spin Echo (SE) sequence and an axial 2D T2W SE sequence (Fig. 2). All MRI sequence parameters can be found in Table 1.

**Table 1:** MRI sequence parameters

Siemens Avanto.fit 1.5 T				Siemens Skyra 3.0 T			Paramed MROpen 0.5 T	
	*T1W TSE*	*T2W TSE*	*T2W TSE*	*T1W STIR*	*T2W TSE*	*T2W TSE*	*T2W Spin Echo*	*T2W Spin Echo*
Orientation	Sagittal	Sagittal	Axial	Sagittal	Sagittal	Axial	Sagittal	Axial
Fat suppression	None	DIXON	None	None	DIXON	None	None	None
Repetition time, TR	400 ms	4090–4190 ms	3100 ms	2000 ms	4100 ms	4100 ms	3659–4147 ms	2880–3994 ms
Echo time, TE	11 ms	92 ms	71 ms	9.2 ms	81 ms	81 ms	120–129 ms	120–129 ms
Inversion time	-	-	-	900 ms	-	-	-	-
Field of view (PxF)	300 × 300 mm^2^	240 × 240 mm^2^	240 × 240 mm^2^	300 × 300 mm^2^	300 × 300 mm^2^	200 × 200 mm^2^	240–300 × 300 mm^2^	240–300 × 300 mm^2^
Matrix size (PxF)	384 × 384	307 × 384	240 × 320	224 × 320	269 × 384	256 × 320	300–372 × 300	240–300 × 240
Slice thickness	4 mm	4 mm	4 mm	3 mm	3 mm	4 mm	4 mm	4 mm
Flip angle	150°	150°	150°	120°	150°	120°	90°	90°
Echo train length	3	16	13	6	17	17	1	1
Pixel bandwidth	155 Hz	260 Hz	170 Hz	270 Hz	360 Hz	250 Hz	UA	UA
NSA	2	2	2	2	3	2	1	1
Slice spacing	4.4 mm	4.4 mm	4.4 mm	3.3 mm	3.3 mm	4.4 mm	4.8 mm	4.8 mm

*STIR* short TI inversion recovery, *TSE* turbo spin echo, *P* × *F* phase encoding direction × frequency encoding direction, *UA* unavailable, *NSA* number of signal averages

### Raters, training, and consensus

The interpretation of all images were performed by three raters, selected from the department: a medical radiologist consultant with 30 years of experience in musculoskeletal MRI (rater A); a Ph.D. student with 28 years of clinical and radiography experience and 4 years of MRI experience including 1000 supervised spinal MRI reports (rater B), and a senior researcher with 12 years of clinical research and MRI experience, including 1000 supervised spinal MRI reports (rater C). All raters had experience with reliability studies and diagnostic classification models in diagnostic imaging [10, 13–16].

To ensure consensus regarding the understanding of the diagnostic classification of degenerative MRI findings and differences between supine and upright MRI, an evaluation manual was prepared based on existing literature [17–26]. For training and identifying practical issues in the evaluation process, all three raters independently analyzed and classified 10 MRIs (not included in the reliability study sample) based on the manual. The raters then met for clarification and adjustments to the assessment and coding process. The evaluation manual was adjusted accordingly, and a second set of 5 MRIs was rated independently to adjust for important disagreements and solidify the final version of the manual.

### MRI evaluation and classification of findings

The three raters initially evaluated the three lower lumbar levels: L3/L4, L4/L5, and L5/S1, a total of 177 disc levels, on the supine MRIs for the presence and grading of the following eight degenerative findings using reliable classification methods: spondylolisthesis; scoliosis; annular fissure; disc degeneration; disc contour; nerve root compromise; spinal stenosis; and facet joint degeneration (see classification details in Table 2).

**Table 2:** Classification of diagnostic MRI findings in the supine position

Diagnostic findings	Scale/categories	Definitions
Spondylolisthesis (Meyerding [22])	Ordinal	Defined as slippage of the vertebral body in relation to the one below in: Anterior, posterior or lateral direction.
	0	Normal
Grade I:	1	Displacement of vertebral body < ¼ of vertebral body below.
Grade II:	2	Displacement of vertebral body < ½ of vertebral body below.
Grade III:	3	Displacement of vertebral body < ¾ of vertebral body below.
Grade IV:	4	Displacement of vertebral body < ^4^/4 of vertebral body below.
Disc degeneration (Pfirrmann [17])	Ordinal	For this study, grades I and II are considered normal.
Grade I:	0	Nucleus pulposus is homogenous and has high, bright white signal intensity. Clear distinction of nucleus and annulus. Normal heights of the intervertebral disc.
Grade II:	0	Like grade I, but the nucleus pulposus is inhomogeneous, with or without clear horizontal bands.
Grade III:	1	Nucleus pulposus being inhomogeneous and gray, unclear distinction of the nucleus and annulus, intermediate signal intensity, and normal to slightly decreased intervertebral disc height.
Grade IV:	2	Inhomogeneous, gray to black nucleus pulposus and no distinction between the nucleus and the annulus. The signal intensity is intermediate to hypointense and normal to moderately decreased disc height.
Grade V:	3	Nucleus pulposus is inhomogeneous and black, with hypointense signal intensity and collapsed disk space.
Nerve root compromise (Lee [19])	Ordinal	
Normal:	0	No contact to nerve roots
Contact:	1	Perineural fat obliteration from two opposing sides. No morphologic change (no signs of compression/deformation) of the nerve root.
Contact and deviation:	2	Perineural fat obliteration surrounding the nerve root from four sides. No morphologic change (no compression/deformation) of nerve root.
Compression:	3	Visible nerve root collapse or morphologic change
Spinal stenosis (Lee [19]) Central	Ordinal	
No stenosis:	0	Up to 3 mm disc bulge is considered normal.
Relative stenosis:	1	Reduced space <50%, but still visible fluid signal around the nerve roots.
Absolute stenosis:	2	50% reduction or more of the dural sac area and no visible signal (dark/black) from cerebrospinal fluid around the nerve roots or medulla spinalis.
Lateral recess		
No stenosis:	0	Normal levels of perineural fat.
Relative stenosis:	1	Reduced space, perineural fat obliteration from at least two opposing sides but still visible perineural fat/CSF signal in the recess.
Absolute stenosis:	2	Reduction of the recess to a point where perineural fat signal/CSF signal no longer is visible.
Foraminal		
No stenosis:	0	Normal upside-down pear shape contour of the foramina with an apical nerve root location.
Relative stenosis:	1	Reduced space, but still visible perineural fat signal in the foramen.
Absolute stenosis:	2	Reduction of the foramen to the point where perineural fat signal is no longer visible.
Facet degeneration		
(Ross/Moore [35]; Pathria [36])	Ordinal	
No degeneration:	0	Normal
Mild degeneration:	1	Mild joint space narrowing and joint irregularity.
Moderate degeneration:	2	Moderate joint space narrowing/irregularity, subchondral sclerosis/osteophyte formation.
Severe degeneration:	3	Little, if any, joint space, severe subchondral sclerosis/ osteophyte formation. Possible subluxation and/or subchondral cyst formation.
Scoliosis (Cobb [24])	Binominal	Defined as any spinal curvature with Cobb's angle greater than 10 degrees.
sinistro convex:	0/1	Apex of the curvature to the left.
dextro convex:	0/1	Apex of the curvature to the right.
rotational:	0/1	Pedicles and spinous process oriented to the left or right.
Annular Fissure (April [18])	Binominal	
	0/1	High T2 signal (HIZ) in the otherwise low signal annulus. Diameter > 1.5 mm. Annulus material visible all around the fissure.
Disc contour (Fardon [26])	Nominal	
Normal or bulge:	0	<3 mm and >25% of the disc periphery (90 degrees). Negative for herniation.
Protrusion:	1	<25% (90 degrees) of disc periphery, distance between disco-vertebral corners is greater than distance of disc material past the base, measured in same plane.
Extrusion:	2	Dimension of disc material in any one direction is greater than distance between disco-vertebral corners. Migration cephalad or caudad indicates extrusion.
Sequestration:	3	Disc material has lost continuity with the parent disc.
Combination of types:	4	Combined protrusion and extrusion

The inter-rater reliability of reporting MRI findings in the supine position has been reported separately and ranged from (Gwet’s AC_1_ or AC_2_) 0.64 to 0.99 [10]. According to probabilistic benchmarking to the Landis and Koch scale, this is equivalent to moderate to almost perfect reliability.

**Fig. 3:**
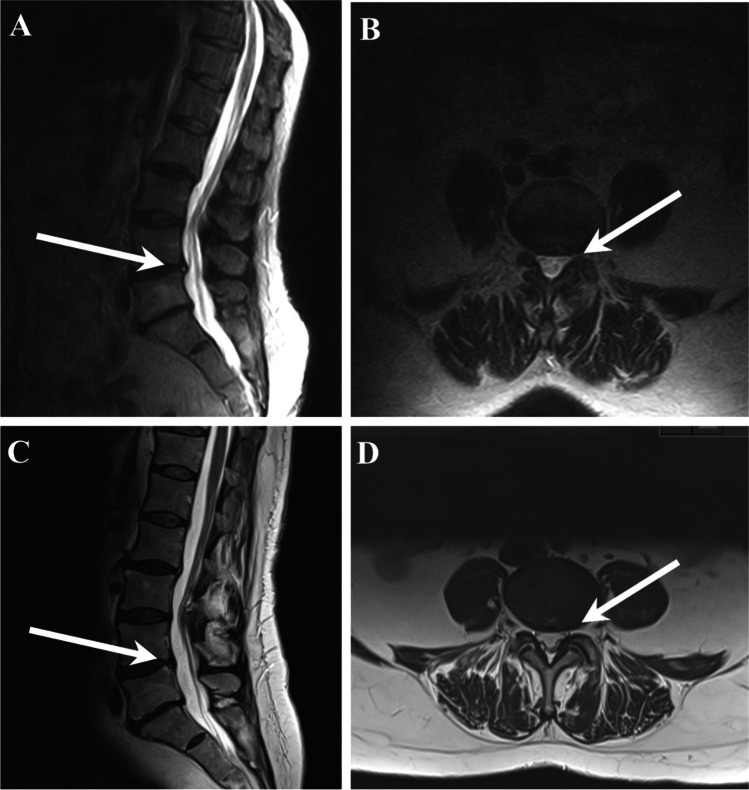
Positional difference of a disc herniation (L4/L5) from supine to upright position. **A** Upright position, sagittal view. **B** Upright position, axial view. **C** Supine position, sagittal view. **D** Supine position, axial view

The same raters then classified observed differences in findings by comparing images obtained in the supine position to images in the upright position into one of five categories: “No change,” and for positional- or grade-type differences classified into “Appeared,” “Disappeared,” “Worsened,” or “Improved” based on validated methods described in the literature [27] (Fig. 3).

The raters retrieved images in PACS (Picture, Archiving and Communication System: Agfa Impax, version 5.2) and filled in the standardized research evaluation form in REDCap. All images were assessed and analyzed on diagnostic Agfa Impax workstations with high-resolution diagnostic monitors (Totoku Monochrome MS33I2_Pair, 3 million pixels, Barco MDNC-2121 color pair, 2 million pixels, and Barco MDNC-2121 monochrome pair, 2 million pixels). The raters worked independently and were blinded with respect to clinical information and the imaging reports.

### Sample size

In a test for agreement between two raters using the Kappa statistic, a sample size of 51 subjects achieves 80% power to detect a true Kappa value of 0.70 in a test of H0: Kappa = κ0 vs. H1: Kappa ≠ κ0, when there are six categories with frequencies equal to 0.48, 0.28, 0.20, 0.03, 0.01, and 0.00. This power calculation was based on a significance level of 0.05 [28].

We decided to include a reasonable sample of 59 individuals, assuming three disc-levels per participant yielding 177 independent units of observation.

### Data management and statistical analysis

Data analysis was carried out in Stata, ver.15.1 (StataCorp LLC, 4905 Lakeway Drive, College Station, Texas 77,845, USA) and AgreeStat 2015.1 for Excel Windows/Mac (Advanced Analytics, LLC. PO Box 2696, Gaithersburg, MD 20,886–2696, USA).

In the statistical analysis, inter-rater reliability was determined for nominal data by calculating percent agreement, and a change corrected agreement coefficient: Gwet’s AC_1_ (unweighted) and AC_2_ (weighted) for respectively pair-wise raters and for three raters overall [29]. Percent agreement and chance-corrected agreement coefficients (except for marginal totals) were reported with 95% confidence intervals. Proportions of absolute agreement were calculated to evaluate the precision of the strength of reliability. Finally, an additional probabilistic method for benchmarking to an interpretation scale was used and presented as the cumulative probability exceeding 95% for the coefficient to fall into one of the following intervals using the benchmark scale of Landis and Koch: < 0.00 poor; 0.00 to 0.20 slight; 0.21 to 0.40 fair; 0.41 to 0.60 moderate; 0.61 to 0.80 substantial, and 0.81 to 1.00 almost perfect [30]. This method allows for a direct comparison between different agreement coefficients and to what extent they are paradox-resistant, i.e., subject to instability if ratings had very low or very high prevalence.

## Results

### Sample characteristics

The 59 participants had a mean age of 38.1 years (standard deviation (SD) 14.1), 27 (45.8%) were females, 23 (39.0%) had LBP only, 12 (20.3%) had LBP and leg pain, and 35 (59.3%) had experienced their symptoms for longer than 4 weeks, and 24 (40.7%) did not have LBP. Participant characteristics are presented in Table 3.

**Table 3:** Characteristics of participants

Characteristics	Cross-sectional study population	Reliability study sample
	(*N* = 230)	(*n* = 59)
Age, in years, mean	42.1 (SD 12.1)	38.1 (SD 14.1)
Females, *n* (%)	118 (51.1%)	27 (45.8%)
Patients, LBP, *n* (%)	72 (31.3%)	23 (39.0%)
Patients, LBP + leg pain, *n* (%)	96 (41.7%)	12 (20.3%)
Symptoms > 4 weeks, *n* (%)	168 (73.0%)	35 (59.3%)
No LBP persons, *n* (%)	62 (27.0%)	24 (40.7%)

### Numbers of supine diagnostic MRI findings at disc level

The total number of positive diagnostic findings based on the supine MRI evaluation (presence of degenerative pathology) for rater A was 270 (9.0% of 3009 ratings per rater), rater B was 375 (12.5%), and rater C was 453 (15.1%) (see Table 4).

**Table 4:** Absolute frequencies of positive diagnostic findings in the supine position and the type of differences observed comparing supine and upright MRI

Observed differences comparing supine and upright MRI Numeric account	Rater A		Rater B		Rater C		Total	
	Supine	Upright	Supine	Upright	Supine	Upright	Supine	Upright
^**1**^ **Spondylolisthesis**								
Positive diagnostic findings supine	4		4		7		15	
Finding appeared upright		0		0		0		0
Finding disappeared upright		0		0		1		1
Finding worsened upright		0		1		2		3
Finding improved upright		0		0		0		0
**Total**		**0**		**1**		**3**		**4**
^**2**^ **Scoliosis**								
Positive diagnostic findings supine	5		3		5		13	
Finding appeared upright		1		3		3		7
Finding disappeared upright		0		0		0		0
Finding worsened upright		1		1		0		2
Finding improved upright		0		0		0		0
**Total**		**2**		**4**		**3**		**9**
^**3**^ **Annular fissure**								
Positive diagnostic findings supine	14		33		29		76	
Finding appeared upright		0		2		3		5
Finding disappeared upright		1		4		5		10
Finding worsened upright		1		0		1		2
Finding improved upright		3		0		0		3
**Total**		**5**		**6**		**9**		**20**
^**3**^ **Disc degeneration**								
Positive diagnostic findings supine	48		72		79		199	
Finding appeared upright		0		0		0		0
Finding disappeared upright		0		0		0		0
Finding worsened upright		0		1		2		3
Finding improved upright		2		0		0		2
**Total**		**2**		**1**		**2**		**5**
^**4**^ **Disc contour**								
Positive diagnostic findings supine	33		48		76		157	
Finding appeared upright		1		5		5		11
Finding disappeared upright		0		1		0		1
Finding worsened upright		0		9		20		29
Finding improved upright		0		0		1		1
**Total**		**1**		**15**		**26**		**42**
^**3**^ **Nerve compromise**								
Positive diagnostic findings supine	19		26		42		87	
Finding appeared upright		0		4		6		10
Finding disappeared upright		0		0		0		0
Finding worsened upright		2		5		2		9
Finding improved upright		1		0		0		1
** Total**		**3**		**9**		**8**		**20**
^**5**^ **Spinal stenosis**								
Positive diagnostic findings supine	26		79		84		189	
Finding appeared upright		0		12		16		28
Finding disappeared upright		0		0		0		0
Finding worsened upright		1		19		9		29
Finding improved upright		4		1		2		7
**Total**		**5**		**32**		**27**		**64**
^6^ **Facet degeneration**								
Positive diagnostic findings supine	121		110		131		362	
Finding appeared upright		0		3		2		5
Finding disappeared upright		0		0		0		0
Finding worsened upright		0		0		0		0
Finding improved upright		0		0		0		0
**Total**		**0**		**3**		**2**		**5**
**Total no. of positive findings supine**	**270**		**375**		**453**		**1098**	
Finding appeared upright		**2**		**29**		**35**		**66**
Finding disappeared upright		**1**		**5**		**6**		**12**
Finding worsened upright		**5**		**36**		**36**		**77**
Finding improved upright		**10**		**1**		**3**		**14**
**Total number of differences upright**		**18**		**71**		**80**		**169**

^1^Fifty-nine subjects × 3 disc levels × 3 directions(ant/retro/lat) = 531 observations

^2^Fifty-nine subjects × 3 (sinistro/dextro/rotational) = 177 observations

^3^Fifty-nine subjects × 3 disc levels = 177 observations

^4^Fifty-nine subjects × 3 disc levels × 2 (bulge+herniation type) = 354 observations

^5^Fifty-nine subjects × 3 disc levels × 5 sites (central, L+R foraminal, L+ R lat. recess) = 885 observations

^6^Fifty-nine subjects × 3 disc levels × 2 (L+R facet joint) × 1 facet orientation/angulation = 531 observations

Total number of observations for all (8) degenerative findings (positive and negative) = 3009/rater

### Number of differences from supine to upright MRI findings at disc level

When comparing all 177 disc levels for observed differences between supine and upright MRI overall, 169 differences were recorded by the three raters (Table 4). Of these, 77 (45.6%) differences in findings were categorized as worsened; 66 (39.0%) appeared, i.e., were not visible on the supine MRI; 12 (7.1%) disappeared, and 14 (8.3%) improved. The number of differences observed per rater across all diagnostic observations were 18 for rater A, 71 for rater B, and 80 for rater C. Summarized, this amount to an average of 56 (15.4%) observed differences on upright MRI per rater, out of 366 observed positive findings on supine MRI.

Proportions of difference in findings from supine to upright position were the following: Nine observed upright differences out of 13 supine findings for scoliosis (0.69), sixty-four observed upright differences out of 189 supine findings of spinal stenosis (0.40), four upright differences out of 15 supine findings of spondylolisthesis (0.27), forty-two upright differences out of 157 supine findings of disc contour (0.27), twenty upright differences out of 76 supine findings of annular fissure (0.26), twenty upright differences out of 87 supine findings of nerve compromise (0.23), and finally, five upright differences out of 199 supine findings of disc degeneration (0.03), and five upright differences out of 362 supine findings of facet degeneration (0.02).

Out of the four sub-categories of observed differences, rater A used “Improved” most often (10 ratings or 55.6% of all observed differences), whereas raters B and C used “Worsened” the most (rater B, 36 ratings or 50.7% and rater C also 36 ratings or 45.0%), followed by “Appeared” (rater B, 29 ratings or 40.8% and rater C, 35 ratings or 43.8%).

### Inter-rater reliability of differences and types of difference comparing supine and upright MRI

The reliability of observed difference and type of difference from supine to upright MRI was almost perfect for individual findings, ranging from Gwet’s AC_1_ = 0.910 for disc contour to 0.998 for spondylolisthesis among individual rater-pairs (Table 5). Overall reliability for the eight degenerative spinal findings was almost perfect (Gwet’s AC_2_ = 0.966; range: 0.929–0.996). Overall, the variability among rater-pairs was low.

**Table 5:** Inter-rater reliability and absolute agreement of observed difference and type of difference from supine to upright MRI at spinal level (nominal scale)

Diagnostic findings (*N* = 177 disc-levels)	Rater A vs. B		Rater A vs. C		Rater B vs. C		All (Gwet’s AC_2_)	Probabilistic benchmarking to Landis and Koch scale
		95% C.I		95% C.I		95% C.I		
Spondylolisthesis								
Gwet’s AC_1_ %-agreement	0.998 99.8	[0.994:1.000]	0.999.4 99.4	[0.988:1.000]	0.996 99.6	[0.991:1.000]	(0.996) 99.6	Almost perfect Almost perfect
Scoliosis								
Gwet’s AC_1_ %-agreement	0.978 96.6	[0.900:0.974]	0.972 97.2	[0.947:0.997]	0.960 96.1	[0.931:0.990]	(0.966) 96.6	Almost perfect Almost perfect
Annular fissure								
Gwet’s AC_1_ %-agreement	0.937 93.8	[0.646:0.948]	0.931 93.2	[0.892:0.970]	0.925 92.7	[0.885:0.965]	(0.931) 93.2	Almost perfect Almost perfect
Disc degeneration								
Gwet’s AC_1_ %-agreement	0.983 98.3	[0.964:1.000]	0.977 97.7	[0.955:1.000]	0.983 98.3	[0.964:1.000]	(0.981) 98.1	Almost perfect Almost perfect
Disc contour								
Gwet’s AC_1_ %-agreement	0.954 95.5	[0.932:0.977]	0.925 92.7	[0.897:0.953]	0.910 91.2	[0.879:0.941]	(0.930) 93.1	Almost perfect Almost perfect
Nerve compromise								
Gwet’s AC_1_ %-agreement	0.931 93.2	[0.892:0.970]	0.943 94.4	[0.907:0.978]	0.913 91.5	[0.870:0.957]	(0.929) 93.0	Almost perfect Almost perfect
Spinal stenosis								
Gwet’s AC_1_ %-agreement	0.959 95.9	[0.946:0.972]	0.968 96.8	[0.956:0.980]	0.946 94.7	[0.931:0.961]	(0.958) 95.8	Almost perfect Almost perfect
Facet degeneration								
Gwet’s AC_1_ %-agreement	0.994 99.4	[0.988:0.998]	0.996 99.6	[0.991:1.000]	0.991 99.1	[0.982:0.999]	(0.994) 99.4	Almost perfect Almost perfect

## Discussion

In this study sample, taken from a cross-sectional study of participants with and without LBP, we wanted to estimate the inter-rater reliability and absolute agreement of a method identifying presence/absence of differences and types of differences in degenerative MRI findings of the lumbar spine compared in the supine and upright position. The three raters independently evaluated 177 disc levels in 59 participants (for scoliosis, the lumbar spine as one unit). Differences from supine to upright MRI were most often observed in relation to scoliosis, spinal stenosis (central-, lateral recess-, and foraminal-stenosis combined), spondylolisthesis, and disc contour. We attribute this to a relationship between disc degeneration and stenosis, causing the ligamentum flavum to become slack, and presenting as a morphologic change in the weight-bearing position. Scoliosis and spondylolisthesis have been considered a gravity-dependent pathologies, also seen on upright radiographs. The least common degenerative pathologies to show changes from supine to upright position were disc degeneration and facet joint degeneration. The most common pathology to disappear in the upright position was annular fissures. It is generally believed that this can be due to (1) the weightbearing position squeezes the inflammatory fluid away from the lesion, or (2) the dural sac is expanded in the lower portion of the low back, due to spinal fluid collection in the upright position. This can also have an effect by squeezing the inflammatory fluid collection in the disc lesion if located posteriorly 

### Statistical considerations

Low prevalence rates of positive findings and high prevalence rates of negative findings exposed this study to the Kappa paradoxes. Therefore, we used Gwet’s AC_1_ and AC_2_ as being more stable chance-corrected agreement coefficients and better suited for our data [29, 31]. In the Reference section, a link can be found for further information on the probabilistic benchmarking method to the Landis and Koch scale proposed by Kilim L. Gwet [32], making our results comparable to other studies using Kappa statistics and the Landis and Koch scale.

### Strength of this study

We think this is an interesting and important topic, and there is a need to establish a more solid piece of evidence to answer whether upright MRI is beneficial for revealing degenerative disease not shown on supine MRI. Also, we found it useful to evaluate if three raters could produce reliable independent readings, not based on consensus. We are in full agreement with Hansen et al., who pointed out, that general high reliability is carried by many patients with no difference between supine and upright position. The grading of fewer patients with differences on the upright MRI is therefore less reliable. This difference can nevertheless be of great importance for the final diagnosis in this group of patients [11]. Maybe most important is the fact that despite the relative lower proportion of MRI findings in this study sample, we found a high proportion of positional differences in the upright position, when a positive degenerative finding was encountered on supine MRI. If disc- and facet joint-degeneration is excluded (0.03 and 0.02 respectively), proportions of positional change ranged from 0.23 to 0.69. These findings are important to research in this field moving forward when designing larger reliability studies with samples of more chronic patients.

Inter-rater reliability and agreement for three raters were found overall acceptable according to Gwet’s Agreement Coefficients for classification of differences and types of differences comparing supine and upright MRI of the lumbar spine (Gwet AC_2_ ranged 0.929–0.996). An acceptable level of reliability indicates that this classification may be applied by experienced health care professionals or researchers in clinical practice, quality assurance, and research.

This study sample was enrolled consecutively from a comparative diagnostic test accuracy study using a fully paired design (all participants received both index tests A and B), which is considered the most robust with respect to bias [33, 34]. Another strength of this study is that the results reflect a genuine study population of referrals from primary care, and the inclusion of controls (no-LBP participants) is valuable. This was required for validating the method used in our following diagnostic test accuracy study.

Also, there is a lack of studies in the literature of reported reliability on methods to determine differences in degenerative MRI findings of the lumbar spine comparing the supine and upright positions. We have identified only one study by Hansen et al. [11] performing a comprehensive reliability study of differences in degenerative findings observed from supine to upright lumbar MRI. They found differences in only 0.5–1.3% of disc-levels including no differences for disc protrusions and extrusions, and they also found considerable variation in the number of differences recorded between raters evaluating 224 disc levels (*n* = 56 LBP patients): A total of 17, 39, and 53 differences for readers A, B, and C respectively, with an average absolute agreement of 97.6%; and for comparison, we found a total of 18, 71, and 80 differences in evaluations of 177 disc levels (*n* = 59 participants) comparing supine and upright MRI for raters A, B, and C respectively, with an average absolute agreement of 96.6%.

**Fig. 4:**
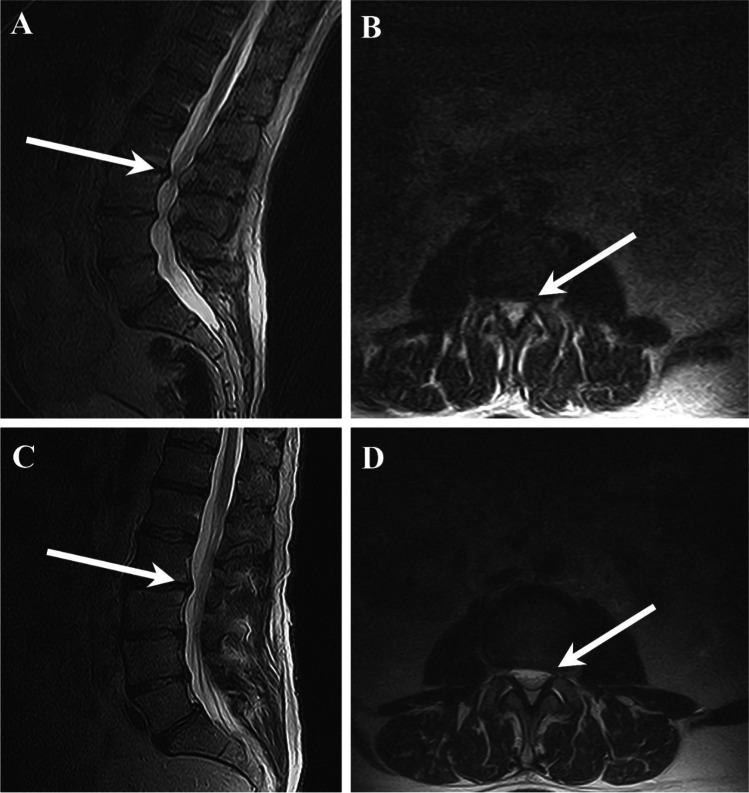
Positional difference of spinal stenosis (L2/L3) from supine to upright position. **A** Upright position, sagittal view. **B** Upright position, axial view. **C** Supine position, sagittal view. **D** Supine position, axial view

Alyas et al. [1], in a pictorial review, concluded that clinically relevant spinal canal stenosis, cauda equina, and nerve root compression might be uncovered by imaging in the erect posture without specifying the type of expected change. Our results differ from some previous studies with respect to the prevalence of differences observed from supine to upright position. In a large retrospective two-rater-study of 4305 LBP patients, Splendiani et al. [2] found differences in 66.6% of the participants from supine to upright MRI when evaluating for types of herniated discs, spinal canal stenosis, lumbar segmental transitional movements, and postural abnormalities of the lumbar spine. In 11%, disc protrusions appeared only on upright MRI. Inter-rater agreement was substantial to almost perfect, with *κ* values ranging from 0.62 to 0.88. We achieved almost perfect agreement (AC_2_ ranging from 0.929 to 0.996), although not easily compared to Splendiani et al., because they did not report percent agreement and reported prevalences, where we, like Hansen et al., reported frequency distributions of the MRI outcomes and positional differences according to the rater (Fig. 4).

### Limitations

All no-LBP persons received supine MRI procedures in a 0.5-T open MRI unit. Raters could possibly identify no-LBP persons since the image quality was lower, and certain sequences were used specifically for the 0.5-T unit. For the 1.5 T and 3 T systems, the sagittal T2-weighted sequence also included T2 fatsat (DIXON). A possible source of bias was that raters could be inclined to observe fewer positive findings in the no-LBP group. However, our primary aim in this study was related to reliability of reporting change between the two positions rather than the presence of MRI degenerative findings, and we believe the unblinding was less likely to impact this outcome. Rater A seemed to have a higher threshold for detecting change compared to raters B and C. The same was reported by Hansen et al. [1]) in their reliability study of upright MRI findings. In both cases, the most experienced rater had a higher threshold. We assume it represents the routines in a busy radiology department, where radiologists most commonly are looking for the presence of pathology that may change management of the patients. In clinical practice of spinal MRI and LBP, these changes generally need to be pronounced and larger than what was seen in this study. Therefore, we concluded that future studies that involve the evaluation of more discrete changes should emphasize training in agreeing on items relevant for the study aims instead of focusing on “normal” radiology procedures.

This study was initiated to investigate the reliability of a three rater analysis to be used in our main study of larger scale. We also included healthy participants, for the sample to resemble the main study for the same reason. Patient numbers might be considered to be low; however, the included number of participants in our study (59) is comparable to other reliability studies. In fact, we identified only one other reliability study on upright MRI (Hansen et al.), which included 56 participants. The low proportion of differences between supine and upright MRI was a challenge, so we decided to use Gwett’s agreement coefficient (AC1 and AC2), which has proved to be robust, when proportions are very low or very high (see also additional files), and we suggest that future reliability studies focus on more chronic patients and include larger samples.

The raters were not randomly selected, and they worked in the same imaging department. Thus, our results may not generalize to other raters with different training.

Due to ethical considerations concerning stability problems and periodic suboptimal image quality of the upright MRI unit, we had to perform a diagnostic MRI procedure in our conventional 1.5 T or 3.0 T tunnel scanner for all LBP patients. The upright MRI unit was considered an experimental device and was mainly used for research purposes. During this project, we encountered numerous shutdowns due to technical issues. However, despite many delays, we managed to schedule most participants on the same day for both procedures and to obtain an acceptable image quality. The stability issues with the upright MRI unit spanned from abrupt magnet quenches, patient table not working, broken coils to severe image artifacts making the images non-optimal for diagnostic use. These issues caused the upright MRI system to be out of production for extended periods of time. Ideally, no-LBP persons should have had the supine MRI procedure performed in the tunnel scanners. However, the hospital policy did not allow us to use the 1.5 T or 3.0 T scanners for persons without indications for diagnostic imaging. This was due to a generally high workload on the conventional scanners.

### Clinical and research implications

Reliability studies are rater and population dependent, and therefore these results may not apply to all the settings and populations where upright MRI technology is used. An acceptable level of reliability carried by almost perfect reliability of negative findings indicates that interpretation and classification of types of differences is difficult and should be used with caution. However, clinically, it is of concern that inter-rater agreement of categorizing positive positional differences is lower. Similar conclusions have been made in another recent study [11]. In recent narrative reviews, Baker et al., Botchu et al., and Michelini et al., concluded that the scanning position is important in the outcome of the MRI examination of the lumbar spine and can be a complementary investigation when there are negative results in conventional MRI in symptomatic patients [6–8].

In conclusion, inter-rater reliability according to Gwet’s agreement coefficients for classification of positive and negative findings for eight degenerative pathologies of the lumbar spine comparing supine and upright MRI scans using three raters was found to be overall acceptable. The raters in this study were in good agreement on the classification of negative and positive findings, but less so for the classification of types of changes between supine and upright positions. The classifications used in this study may be sufficiently comprehensible to be applied by health care professionals and in clinical practice, quality assurance, and research, but while the sample size seemed reasonable, our results were driven by a high reliability of the many negative findings. Larger studies or studies including carefully selected patients with specific (and more chronic) degenerative pathologies are needed to investigate the reliability of changes in findings comparing supine and upright MRI. Upright MRI is a supplementary modality that may offer a diagnostic imaging solution in situations where inconsistency is found between clinical findings, patient symptoms, and conventional MRI. It is noteworthy, that in our study, 85% of identified changes between supine and upright position were either not seen or underestimated on supine MRI. The remaining 15% of changes was only seen on supine MRI or underestimated on upright MRI.

## Supplementary Information

Below is the link to the electronic supplementary material.

Supplementary file1 (DOCX 39 KB)

Supplementary file2 (DOCX 35 KB)

Supplementary file3 (DOCX 38 KB)

Supplementary file4 (DOCX 38 KB)

## Data Availability

The datasets used and/or analyzed during the current study are available from the corresponding author on reasonable request.

## Abbreviations

MRI: Magnetic resonance imaging
AC1: Agreement coefficient (unweighted, two raters)
AC2: Agreement coefficient (weighted, more than two raters)
LBP: Low back pain
REDCap: Research Electronic Data Capture program
PACS: Picture archiving and communication system (i.e. Agfa Impax)
CSF: Cerebrospinal fluid

## Declarations

### Authors’ information

Information on authors’ affiliations is found on the first page of this article.
