# Supplementary material for: Reliability of reporting differences in degenerative MRI findings of the lumbar spine from the supine to the upright position
Source: Skeletal Radiol. 2022 May 10;51(11):2141–54. doi: 10.1007/s00256-022-04060-2 (PMC9463326; doi:10.1007/s00256-022-04060-2)
Supplement: Supplementary file 1 — Supplementary file1 (DOCX 39 KB) [file 256_2022_4060_MOESM1_ESM.docx]

| **Inter-rater reliability of observed differences comparing** |
| --- |
| **degenerative MRI-findings in the supine and upright positions** |
| **AgreeStat 2015.6.1** |
| MODULE: Two-Rater Chance-Corrected Agreement Coefficients (Time: 16:52:24. Date: 14. juli 2020) |

**Group: MR02 Spondylolisthesis changetype**

Rater 1

|  | **0** | **1** | **2** | **3** | **4** | **Missing** | **Total** |  |
| --- | --- | --- | --- | --- | --- | --- | --- | --- |
| **0** | 530 | 0 | 0 | 1 | 0 | 0 | 531 | [100%] |
| **1** | 0 | 0 | 0 | 0 | 0 | 0 | 0 | [0%] |
| **2** | 0 | 0 | 0 | 0 | 0 | 0 | 0 | [0%] |
| **3** | 0 | 0 | 0 | 0 | 0 | 0 | 0 | [0%] |
| **4** | 0 | 0 | 0 | 0 | 0 | 0 | 0 | [0%] |
| **Missing** | 0 | 0 | 0 | 0 | 0 | 0 | 0 | [0%] |
| **Total** | 530 | 0 | 0 | 1 | 0 | 0 | 531 | [100%] |
| [99,8%] | | [0%] | [0%] | [0,2%] | [0%] | [0%] | [100%] |  |

DISTRIBUTION OF SUBJECTS BY RATER AND CATEGORY (0=No change, 1=Appeared, 2=Disappeared; 3=Worsened and 4=Improved) Rater 2

## INTER-RATER RELIABILITY COEFFICIENTS AND ASSOCIATED PRECISION MEASURES

Unweighted Agreement Coefficients

| METHOD | **Coeff.** | **StdErr** | **95% C.I.** | **p-Value** |
| --- | --- | --- | --- | --- |
| **Cohen's Kappa** | 0,00000 | 5,47794E-17 | 0 to 0 | n/a |
| **Gwet's AC_1_** | 0,99812 | 0,001885011 | 0,994 to 1 | 0,000E+00 |
| **Scott's Pi** | -0,00094 | 0,000943395 | -0,003 to 0,001 | 3,182E-01 |
| **Krippendorff's Alpha** | 0,00000 | 0,000943395 | -0,002 to 0,002 | 1,000E+00 |
| **Brenann-Prediger** | 0,99765 | 0,002354049 | 0,993 to 1 | 0,000E+00 |
| **Percent Agreement** | 0,99812 | 0,001883239 | 0,994 to 1 | 0,000E+00 |

# LANDIS-KOCH INTERPRETATION OF THE AGREEMENT COEFFICIENTS

Benchmarking Unweighted Agreement Coefficients using Cumulative Membership Probabilities

| **Benchmark** | **Interpretation** | **Cohen** | **Gwet** | **Scott's** | **Krippendorff** | **Brennan** | **Percent** |
| --- | --- | --- | --- | --- | --- | --- | --- |
| **Scale** |  | **Kappa** | **AC_1_** | **Pi** | **Alpha** | **Prediger** | **Agreement** |
| 0,8 to 1 | Almost Perfect | 0,00000 | 1,00000 | 0,00000 | 0,00000 | 1,00000 | 1,00000 |
| 0,6 to 0,8 | Substantial | 0,00000 | 1,00000 | 0,00000 | 0,00000 | 1,00000 | 1,00000 |
| 0,4 to 0,6 | Moderate | 0,00000 | 1,00000 | 0,00000 | 0,00000 | 1,00000 | 1,00000 |
| 0,2 to 0,4 | Fair | 0,00000 | 1,00000 | 0,00000 | 0,00000 | 1,00000 | 1,00000 |
| 0 to 0,2 | Slight | 0,50000 | 1,00000 | 0,15888 | 0,50000 | 1,00000 | 1,00000 |
| Less than 0 | Poor | 1,00000 | 1,00000 | 1,00000 | 1,00000 | 1,00000 | 1,00000 |

**Group: MR05 Scoliosis changetype**

Rater 1

|  | **0** | **1** | **2** | **3** | **4** | **Missing** | **Total** |  |
| --- | --- | --- | --- | --- | --- | --- | --- | --- |
| **0** | 171 | 3 | 0 | 1 | 0 | 0 | 175 | [98,9%] |
| **1** | 1 | 0 | 0 | 0 | 0 | 0 | 1 | [0,6%] |
| **2** | 0 | 0 | 0 | 0 | 0 | 0 | 0 | [0%] |
| **3** | 1 | 0 | 0 | 0 | 0 | 0 | 1 | [0,6%] |
| **4** | 0 | 0 | 0 | 0 | 0 | 0 | 0 | [0%] |
| **Missing** | 0 | 0 | 0 | 0 | 0 | 0 | 0 | [0%] |
| **Total** | 173 | 3 | 0 | 1 | 0 | 0 | 177 | [100%] |
| [97,7%] | | [1,7%] | [0%] | [0,6%] | [0%] | [0%] | [100%] |  |

DISTRIBUTION OF SUBJECTS BY RATER AND CATEGORY (0=No change, 1=Appeared, 2=Disappeared; 3=Worsened and 4=Improved) Rater 2

## INTER-RATER RELIABILITY COEFFICIENTS AND ASSOCIATED PRECISION MEASURES

Unweighted Agreement Coefficients

| METHOD | **Coeff.** | **StdErr** | **95% C.I.** | **p-Value** |
| --- | --- | --- | --- | --- |
| **Cohen's Kappa** | -0,01143 | 0,005890722 | -0,023 to 0 | 5,397E-02 |

| **Gwet's AC_1_** | 0,96582 | 0,013870432 | 0,938 to 0,993 | 4,955E-130 |
| --- | --- | --- | --- | --- |
| **Scott's Pi** | -0,01336 | 0,005561369 | -0,024 to -0,002 | 1,734E-02 |
| **Krippendorff's Alpha** | -0,01050 | 0,005561369 | -0,021 to 0 | 6,076E-02 |
| **Brenann-Prediger** | 0,95763 | 0,017051166 | 0,924 to 0,991 | 2,620E-114 |
| **Percent Agreement** | 0,96610 | 0,013640933 | 0,939 to 0,993 | 2,765E-131 |

# LANDIS-KOCH INTERPRETATION OF THE AGREEMENT COEFFICIENTS

Benchmarking Unweighted Agreement Coefficients using Cumulative Membership Probabilities

| **Benchmark** | **Interpretation** | **Cohen** | **Gwet** | **Scott's** | **Krippendorff** | **Brennan** | **Percent** |
| --- | --- | --- | --- | --- | --- | --- | --- |
| **Scale** |  | **Kappa** | **AC_1_** | **Pi** | **Alpha** | **Prediger** | **Agreement** |
| 0,8 to 1 | Almost Perfect | 0,00000 | 1,00000 | 0,00000 | 0,00000 | 1,00000 | 1,00000 |
| 0,6 to 0,8 | Substantial | 0,00000 | 1,00000 | 0,00000 | 0,00000 | 1,00000 | 1,00000 |
| 0,4 to 0,6 | Moderate | 0,00000 | 1,00000 | 0,00000 | 0,00000 | 1,00000 | 1,00000 |
| 0,2 to 0,4 | Fair | 0,00000 | 1,00000 | 0,00000 | 0,00000 | 1,00000 | 1,00000 |
| 0 to 0,2 | Slight | 0,02618 | 1,00000 | 0,00815 | 0,02956 | 1,00000 | 1,00000 |
| Less than 0 | Poor | 1,00000 | 1,00000 | 1,00000 | 1,00000 | 1,00000 | 1,00000 |

**Group: MR06 Annular fissure changetype**

Rater 1

|  | **0** | **1** | **2** | **3** | **4** | **Missing** | **Total** |  |
| --- | --- | --- | --- | --- | --- | --- | --- | --- |
| **0** | 166 | 2 | 4 | 0 | 0 | 0 | 172 | [97,2%] |
| **1** | 0 | 0 | 0 | 0 | 0 | 0 | 0 | [0%] |
| **2** | 1 | 0 | 0 | 0 | 0 | 0 | 1 | [0,6%] |
| **3** | 1 | 0 | 0 | 0 | 0 | 0 | 1 | [0,6%] |
| **4** | 3 | 0 | 0 | 0 | 0 | 0 | 3 | [1,7%] |
| **Missing** | 0 | 0 | 0 | 0 | 0 | 0 | 0 | [0%] |
| **Total** | 171 | 2 | 4 | 0 | 0 | 0 | 177 | [100%] |
| [96,6%] | | [1,1%] | [2,3%] | [0%] | [0%] | [0%] | [100%] |  |

DISTRIBUTION OF SUBJECTS BY RATER AND CATEGORY (0=No change, 1=Appeared, 2=Disappeared; 3=Worsened and 4=Improved) Rater 2

## INTER-RATER RELIABILITY COEFFICIENTS AND ASSOCIATED PRECISION MEASURES

Unweighted Agreement Coefficients

| METHOD | **Coeff.** | **StdErr** | **95% C.I.** | **p-Value** |
| --- | --- | --- | --- | --- |
| **Cohen's Kappa** | -0,01777 | 0,00575125 | -0,029 to -0,006 | 2,325E-03 |
| **Gwet's AC_1_** | 0,93689 | 0,018758648 | 0,9 to 0,974 | 7,223E-106 |
| **Scott's Pi** | -0,02098 | 0,006400104 | -0,034 to -0,008 | 1,262E-03 |
| **Krippendorff's Alpha** | -0,01809 | 0,006400104 | -0,031 to -0,005 | 5,247E-03 |
| **Brenann-Prediger** | 0,92232 | 0,022747349 | 0,877 to 0,967 | 3,308E-91 |
| **Percent Agreement** | 0,93785 | 0,01819788 | 0,902 to 0,974 | 4,104E-108 |

# LANDIS-KOCH INTERPRETATION OF THE AGREEMENT COEFFICIENTS

Benchmarking Unweighted Agreement Coefficients using Cumulative Membership Probabilities

| **Benchmark** | **Interpretation** | **Cohen** | **Gwet** | **Scott's** | **Krippendorff** | **Brennan** | **Percent** |
| --- | --- | --- | --- | --- | --- | --- | --- |
| **Scale** |  | **Kappa** | **AC_1_** | **Pi** | **Alpha** | **Prediger** | **Agreement** |
| 0,8 to 1 | Almost Perfect | 0,00000 | 1,00000 | 0,00000 | 0,00000 | 1,00000 | 1,00000 |
| 0,6 to 0,8 | Substantial | 0,00000 | 1,00000 | 0,00000 | 0,00000 | 1,00000 | 1,00000 |
| 0,4 to 0,6 | Moderate | 0,00000 | 1,00000 | 0,00000 | 0,00000 | 1,00000 | 1,00000 |
| 0,2 to 0,4 | Fair | 0,00000 | 1,00000 | 0,00000 | 0,00000 | 1,00000 | 1,00000 |
| 0 to 0,2 | Slight | 0,00100 | 1,00000 | 0,00052 | 0,00235 | 1,00000 | 1,00000 |
| Less than 0 | Poor | 1,00000 | 1,00000 | 1,00000 | 1,00000 | 1,00000 | 1,00000 |

**Group: MR07 Disc degeneration changetype**

Rater 1

DISTRIBUTION OF SUBJECTS BY RATER AND CATEGORY (0=No change, 1=Appeared, 2=Disappeared; 3=Worsened and 4=Improved) Rater 2

[98,9%]

|  | **0** | **1** | **2** | **3** | **4** | **Missing** | **Total** |
| --- | --- | --- | --- | --- | --- | --- | --- |
| **0** | 174 | 0 | 0 | 1 | 0 | 0 | 175 |
| **1** | 0 | 0 | 0 | 0 | 0 | 0 | 0 |
| **2** | 0 | 0 | 0 | 0 | 0 | 0 | 0 |
| **3** | 0 | 0 | 0 | 0 | 0 | 0 | 0 |

[0%]

[0%]

[0%]

| **4** | 2 | 0 | 0 | 0 | 0 | 0 | 2 | [1,1%] |
| --- | --- | --- | --- | --- | --- | --- | --- | --- |
| **Missing** | 0 | 0 | 0 | 0 | 0 | 0 | 0 | [0%] |
| **Total** | 176 | 0 | 0 | 1 | 0 | 0 | 177 | [100%] |
|  | [99,4%] | [0%] | [0%] | [0,6%] | [0%] | [0%] | [100%] |  |

## INTER-RATER RELIABILITY COEFFICIENTS AND ASSOCIATED PRECISION MEASURES

Unweighted Agreement Coefficients

| METHOD | **Coeff.** | **StdErr** | **95% C.I.** | **p-Value** |
| --- | --- | --- | --- | --- |
| **Cohen's Kappa** | -0,00378 | 0,002675844 | -0,009 to 0,002 | 1,594E-01 |
| **Gwet's AC_1_** | 0,98298 | 0,009811998 | 0,964 to 1 | 3,842E-157 |
| **Scott's Pi** | -0,00664 | 0,003912993 | -0,014 to 0,001 | 9,172E-02 |
| **Krippendorff's Alpha** | -0,00379 | 0,003912993 | -0,012 to 0,004 | 3,339E-01 |
| **Brenann-Prediger** | 0,97881 | 0,012162299 | 0,955 to 1 | 9,212E-141 |
| **Percent Agreement** | 0,98305 | 0,009729839 | 0,964 to 1 | 8,856E-158 |

# LANDIS-KOCH INTERPRETATION OF THE AGREEMENT COEFFICIENTS

Benchmarking Unweighted Agreement Coefficients using Cumulative Membership Probabilities

| **Benchmark** | **Interpretation** | **Cohen** | **Gwet** | **Scott's** | **Krippendorff** | **Brennan** | **Percent** |
| --- | --- | --- | --- | --- | --- | --- | --- |
| **Scale** |  | **Kappa** | **AC_1_** | **Pi** | **Alpha** | **Prediger** | **Agreement** |
| 0,8 to 1 | Almost Perfect | 0,00000 | 1,00000 | 0,00000 | 0,00000 | 1,00000 | 1,00000 |
| 0,6 to 0,8 | Substantial | 0,00000 | 1,00000 | 0,00000 | 0,00000 | 1,00000 | 1,00000 |
| 0,4 to 0,6 | Moderate | 0,00000 | 1,00000 | 0,00000 | 0,00000 | 1,00000 | 1,00000 |
| 0,2 to 0,4 | Fair | 0,00000 | 1,00000 | 0,00000 | 0,00000 | 1,00000 | 1,00000 |
| 0 to 0,2 | Slight | 0,07884 | 1,00000 | 0,04498 | 0,16629 | 1,00000 | 1,00000 |
| Less than 0 | Poor | 1,00000 | 1,00000 | 1,00000 | 1,00000 | 1,00000 | 1,00000 |

**Group: MR08 Disc herniation changetype**

| DISTRIBUTION OF SUBJECTS BY RATER AND CATEGORY (0=No change, 1=Appeared, 2=Disappeared; 3=Worsened and 4=Improved) |
| --- |
| Rater 2 |

Rater 1

|  | **0** | **1** | **2** | **3** | **4** | **Missing** | **Total** |  |
| --- | --- | --- | --- | --- | --- | --- | --- | --- |
| **0** | 338 | 5 | 1 | 9 | 0 | 0 | 353 | [99,7%] |
| **1** | 1 | 0 | 0 | 0 | 0 | 0 | 1 | [0,3%] |
| **2** | 0 | 0 | 0 | 0 | 0 | 0 | 0 | [0%] |
| **3** | 0 | 0 | 0 | 0 | 0 | 0 | 0 | [0%] |
| **4** | 0 | 0 | 0 | 0 | 0 | 0 | 0 | [0%] |
| **Missing** | 0 | 0 | 0 | 0 | 0 | 0 | 0 | [0%] |
| **Total** | 339 | 5 | 1 | 9 | 0 | 0 | 354 | [100%] |
|  | [95,8%] | [1,4%] | [0,3%] | [2,5%] | [0%] | [0%] | [100%] |  |

## INTER-RATER RELIABILITY COEFFICIENTS AND ASSOCIATED PRECISION MEASURES

Unweighted Agreement Coefficients

| METHOD | **Coeff.** | **StdErr** | **95% C.I.** | **p-Value** |
| --- | --- | --- | --- | --- |
| **Cohen's Kappa** | -0,00354 | 0,003349542 | -0,01 to 0,003 | 2,908E-01 |
| **Gwet's AC_1_** | 0,95429 | 0,01130455 | 0,932 to 0,977 | 3,846E-236 |
| **Scott's Pi** | -0,01678 | 0,004250111 | -0,025 to -0,008 | 9,465E-05 |
| **Krippendorff's Alpha** | -0,01535 | 0,004250111 | -0,024 to -0,007 | 3,487E-04 |
| **Brenann-Prediger** | 0,94350 | 0,013820945 | 0,916 to 0,971 | 1,796E-205 |
| **Percent Agreement** | 0,95480 | 0,011056756 | 0,933 to 0,977 | 1,844E-239 |

# LANDIS-KOCH INTERPRETATION OF THE AGREEMENT COEFFICIENTS

Benchmarking Unweighted Agreement Coefficients using Cumulative Membership Probabilities

| **Benchmark** | **Interpretation** | **Cohen** | **Gwet** | **Scott's** | **Krippendorff** | **Brennan** | **Percent** |
| --- | --- | --- | --- | --- | --- | --- | --- |
| **Scale** |  | **Kappa** | **AC_1_** | **Pi** | **Alpha** | **Prediger** | **Agreement** |
| 0,8 to 1 | Almost Perfect | 0,00000 | 1,00000 | 0,00000 | 0,00000 | 1,00000 | 1,00000 |
| 0,6 to 0,8 | Substantial | 0,00000 | 1,00000 | 0,00000 | 0,00000 | 1,00000 | 1,00000 |
| 0,4 to 0,6 | Moderate | 0,00000 | 1,00000 | 0,00000 | 0,00000 | 1,00000 | 1,00000 |
| 0,2 to 0,4 | Fair | 0,00000 | 1,00000 | 0,00000 | 0,00000 | 1,00000 | 1,00000 |
| 0 to 0,2 | Slight | 0,14504 | 1,00000 | 0,00004 | 0,00015 | 1,00000 | 1,00000 |
| Less than 0 | Poor | 1,00000 | 1,00000 | 1,00000 | 1,00000 | 1,00000 | 1,00000 |

**Group: MR09 Nerve compromise changetype**

Rater 1

|  | **0** | **1** | **2** | **3** | **4** | **Missing** | **Total** |  |
| --- | --- | --- | --- | --- | --- | --- | --- | --- |
| **0** | 165 | 4 | 0 | 5 | 0 | 0 | 174 | [98,3%] |
| **1** | 0 | 0 | 0 | 0 | 0 | 0 | 0 | [0%] |
| **2** | 0 | 0 | 0 | 0 | 0 | 0 | 0 | [0%] |
| **3** | 2 | 0 | 0 | 0 | 0 | 0 | 2 | [1,1%] |
| **4** | 1 | 0 | 0 | 0 | 0 | 0 | 1 | [0,6%] |
| **Missing** | 0 | 0 | 0 | 0 | 0 | 0 | 0 | [0%] |
| **Total** | 168 | 4 | 0 | 5 | 0 | 0 | 177 | [100%] |
| [94,9%] | | [2,3%] | [0%] | [2,8%] | [0%] | [0%] | [100%] |  |

DISTRIBUTION OF SUBJECTS BY RATER AND CATEGORY (0=No change, 1=Appeared, 2=Disappeared; 3=Worsened and 4=Improved) Rater 2

## INTER-RATER RELIABILITY COEFFICIENTS AND ASSOCIATED PRECISION MEASURES

Unweighted Agreement Coefficients

| METHOD | **Coeff.** | **StdErr** | **95% C.I.** | **p-Value** |
| --- | --- | --- | --- | --- |
| **Cohen's Kappa** | -0,01773 | 0,008241589 | -0,034 to -0,001 | 3,283E-02 |
| **Gwet's AC_1_** | 0,93106 | 0,01958389 | 0,892 to 0,97 | 2,333E-102 |
| **Scott's Pi** | -0,02534 | 0,007451756 | -0,04 to -0,011 | 8,307E-04 |
| **Krippendorff's Alpha** | -0,02245 | 0,007451756 | -0,037 to -0,008 | 2,974E-03 |
| **Brenann-Prediger** | 0,91525 | 0,023687161 | 0,869 to 0,962 | 6,786E-88 |
| **Percent Agreement** | 0,93220 | 0,018949729 | 0,895 to 0,97 | 8,708E-105 |

# LANDIS-KOCH INTERPRETATION OF THE AGREEMENT COEFFICIENTS

Benchmarking Unweighted Agreement Coefficients using Cumulative Membership Probabilities

| **Benchmark** | **Interpretation** | **Cohen** | **Gwet** | **Scott's** | **Krippendorff** | **Brennan** | **Percent** |
| --- | --- | --- | --- | --- | --- | --- | --- |
| **Scale** |  | **Kappa** | **AC_1_** | **Pi** | **Alpha** | **Prediger** | **Agreement** |
| 0,8 to 1 | Almost Perfect | 0,00000 | 1,00000 | 0,00000 | 0,00000 | 1,00000 | 1,00000 |
| 0,6 to 0,8 | Substantial | 0,00000 | 1,00000 | 0,00000 | 0,00000 | 1,00000 | 1,00000 |

| 0,4 to 0,6 | Moderate | 0,00000 | 1,00000 | 0,00000 | 0,00000 | 1,00000 | 1,00000 |
| --- | --- | --- | --- | --- | --- | --- | --- |
| 0,2 to 0,4 | Fair | 0,00000 | 1,00000 | 0,00000 | 0,00000 | 1,00000 | 1,00000 |
| 0 to 0,2 | Slight | 0,01573 | 1,00000 | 0,00034 | 0,00130 | 1,00000 | 1,00000 |
| Less than 0 | Poor | 1,00000 | 1,00000 | 1,00000 | 1,00000 | 1,00000 | 1,00000 |

**Group: MR12 Spinal stenosis changetype**

Rater 1

|  | **0** | **1** | **2** | **3** | **4** | **Missing** | **Total** |  |
| --- | --- | --- | --- | --- | --- | --- | --- | --- |
| **0** | 849 | 12 | 0 | 18 | 1 | 0 | 880 | [99,4%] |
| **1** | 0 | 0 | 0 | 0 | 0 | 0 | 0 | [0%] |
| **2** | 0 | 0 | 0 | 0 | 0 | 0 | 0 | [0%] |
| **3** | 1 | 0 | 0 | 0 | 0 | 0 | 1 | [0,1%] |
| **4** | 3 | 0 | 0 | 1 | 0 | 0 | 4 | [0,5%] |
| **Missing** | 0 | 0 | 0 | 0 | 0 | 0 | 0 | [0%] |
| **Total** | 853 | 12 | 0 | 19 | 1 | 0 | 885 | [100%] |
| [96,4%] | | [1,4%] | [0%] | [2,1%] | [0,1%] | [0%] | [100%] |  |

DISTRIBUTION OF SUBJECTS BY RATER AND CATEGORY (0=No change, 1=Appeared, 2=Disappeared; 3=Worsened and 4=Improved) Rater 2

## INTER-RATER RELIABILITY COEFFICIENTS AND ASSOCIATED PRECISION MEASURES

Unweighted Agreement Coefficients

| METHOD | **Coeff.** | **StdErr** | **95% C.I.** | **p-Value** |
| --- | --- | --- | --- | --- |
| **Cohen's Kappa** | 0,02156 | 0,025440911 | -0,028 to 0,071 | 3,970E-01 |
| **Gwet's AC_1_** | 0,95890 | 0,006782029 | 0,946 to 0,972 | 0,000E+00 |
| **Scott's Pi** | 0,01241 | 0,026143855 | -0,039 to 0,064 | 6,350E-01 |
| **Krippendorff's Alpha** | 0,01297 | 0,026143855 | -0,038 to 0,064 | 6,199E-01 |
| **Brenann-Prediger** | 0,94915 | 0,008305116 | 0,933 to 0,965 | 0,000E+00 |
| **Percent Agreement** | 0,95932 | 0,006644093 | 0,946 to 0,972 | 0,000E+00 |

# LANDIS-KOCH INTERPRETATION OF THE AGREEMENT COEFFICIENTS

Benchmarking Unweighted Agreement Coefficients using Cumulative Membership Probabilities

| **Benchmark** | **Interpretation** | **Cohen** | **Gwet** | **Scott's** | **Krippendorff** | **Brennan** | **Percent** |
| --- | --- | --- | --- | --- | --- | --- | --- |
| **Scale** |  | **Kappa** | **AC_1_** | **Pi** | **Alpha** | **Prediger** | **Agreement** |
| 0,8 to 1 | Almost Perfect | 0,00000 | 1,00000 | 0,00000 | 0,00000 | 1,00000 | 1,00000 |
| 0,6 to 0,8 | Substantial | 0,00000 | 1,00000 | 0,00000 | 0,00000 | 1,00000 | 1,00000 |
| 0,4 to 0,6 | Moderate | 0,00000 | 1,00000 | 0,00000 | 0,00000 | 1,00000 | 1,00000 |
| 0,2 to 0,4 | Fair | 0,00000 | 1,00000 | 0,00000 | 0,00000 | 1,00000 | 1,00000 |
| 0 to 0,2 | Slight | 0,80162 | 1,00000 | 0,68256 | 0,69012 | 1,00000 | 1,00000 |
| Less than 0 | Poor | 1,00000 | 1,00000 | 1,00000 | 1,00000 | 1,00000 | 1,00000 |

**Group: MR14 Facet joint degeneration chang**

Rater 1

|  | **0** | **1** | **2** | **3** | **4** | **Missing** | **Total** |  |
| --- | --- | --- | --- | --- | --- | --- | --- | --- |
| **0** | 528 | 3 | 0 | 0 | 0 | 0 | 531 | [100%] |
| **1** | 0 | 0 | 0 | 0 | 0 | 0 | 0 | [0%] |
| **2** | 0 | 0 | 0 | 0 | 0 | 0 | 0 | [0%] |
| **3** | 0 | 0 | 0 | 0 | 0 | 0 | 0 | [0%] |
| **4** | 0 | 0 | 0 | 0 | 0 | 0 | 0 | [0%] |
| **Missing** | 0 | 0 | 0 | 0 | 0 | 0 | 0 | [0%] |
| **Total** | 528 | 3 | 0 | 0 | 0 | 0 | 531 | [100%] |
| [99,4%] | | [0,6%] | [0%] | [0%] | [0%] | [0%] | [100%] |  |

DISTRIBUTION OF SUBJECTS BY RATER AND CATEGORY (0=No change, 1=Appeared, 2=Disappeared; 3=Worsened and 4=Improved) Rater 2

## INTER-RATER RELIABILITY COEFFICIENTS AND ASSOCIATED PRECISION MEASURES

Unweighted Agreement Coefficients

| METHOD | **Coeff.** | **StdErr** | **95% C.I.** | **p-Value** |
| --- | --- | --- | --- | --- |
| **Cohen's Kappa** | 0,00000 | 1,13024E-14 | 0 to 0 | 8,268E-02 |
| **Gwet's AC_1_** | 0,99434 | 0,003264883 | 0,988 to 1 | 0,000E+00 |
| **Scott's Pi** | -0,00283 | 0,001637089 | -0,006 to 0 | 8,414E-02 |
| **Krippendorff's Alpha** | -0,00189 | 0,001637089 | -0,005 to 0,001 | 2,492E-01 |

| **Brenann-Prediger** | 0,99294 | 0,004069632 | 0,985 to 1 | 0,000E+00 |
| --- | --- | --- | --- | --- |
| **Percent Agreement** | 0,99435 | 0,003255706 | 0,988 to 1 | 0,000E+00 |

# LANDIS-KOCH INTERPRETATION OF THE AGREEMENT COEFFICIENTS

Benchmarking Unweighted Agreement Coefficients using Cumulative Membership Probabilities

| **Benchmark** | **Interpretation** | **Cohen** | **Gwet** | **Scott's** | **Krippendorff** | **Brennan** | **Percent** |
| --- | --- | --- | --- | --- | --- | --- | --- |
| **Scale** |  | **Kappa** | **AC_1_** | **Pi** | **Alpha** | **Prediger** | **Agreement** |
| 0,8 to 1 | Almost Perfect | 0,00000 | 1,00000 | 0,00000 | 0,00000 | 1,00000 | 1,00000 |
| 0,6 to 0,8 | Substantial | 0,00000 | 1,00000 | 0,00000 | 0,00000 | 1,00000 | 1,00000 |
| 0,4 to 0,6 | Moderate | 0,00000 | 1,00000 | 0,00000 | 0,00000 | 1,00000 | 1,00000 |
| 0,2 to 0,4 | Fair | 0,00000 | 1,00000 | 0,00000 | 0,00000 | 1,00000 | 1,00000 |
| 0 to 0,2 | Slight | 0,04105 | 1,00000 | 0,04178 | 0,12433 | 1,00000 | 1,00000 |
| Less than 0 | Poor | 1,00000 | 1,00000 | 1,00000 | 1,00000 | 1,00000 | 1,00000 |

**Group: Overall**

Rater 1

|  | **0** | **1** | **2** | **3** | **4** | **Missing** | **Total** |  |
| --- | --- | --- | --- | --- | --- | --- | --- | --- |
| **0** | 5835 | 60 | 47 | 35 | 1 | 0 | 5978 | [99,3%] |
| **1** | 18 | 0 | 0 | 0 | 0 | 0 | 18 | [0,3%] |
| **2** | 6 | 0 | 1 | 0 | 0 | 0 | 7 | [0,1%] |
| **3** | 5 | 0 | 0 | 0 | 0 | 0 | 5 | [0,1%] |
| **4** | 9 | 0 | 0 | 1 | 0 | 0 | 10 | [0,2%] |
| **Missing** | 0 | 0 | 0 | 0 | 0 | 0 | 0 | [0%] |
| **Total** | 5873 | 60 | 48 | 36 | 1 | 0 | 6018 | [100%] |
| [97,6%] | | [1%] | [0,8%] | [0,6%] | [0%] | [0%] | [100%] |  |

DISTRIBUTION OF SUBJECTS BY RATER AND CATEGORY (0=No change, 1=Appeared, 2=Disappeared; 3=Worsened and 4=Improved) Rater 2

| INTER-RATER RELIABILITY COEFFICIENTS AND ASSOCIATED PRECISION MEASURES |
| --- |
| Unweighted Agreement Coefficients |

| METHOD | **Coeff.** | **StdErr** | **95% C.I.** | **p-Value** |
| --- | --- | --- | --- | --- |
| **Cohen's Kappa** | 0,00963 | 0,01189581 | -0,014 to 0,033 | 4,183E-01 |
| **Gwet's AC_1_** | 0,96953 | 0,002241373 | 0,965 to 0,974 | 0,000E+00 |
| **Scott's Pi** | 0,00614 | 0,012000057 | -0,017 to 0,03 | 6,087E-01 |
| **Krippendorff's Alpha** | 0,00623 | 0,012000057 | -0,017 to 0,03 | 6,039E-01 |
| **Brenann-Prediger** | 0,96220 | 0,002759695 | 0,957 to 0,968 | 0,000E+00 |
| **Percent Agreement** | 0,96976 | 0,002207756 | 0,965 to 0,974 | 0,000E+00 |

# LANDIS-KOCH INTERPRETATION OF THE AGREEMENT COEFFICIENTS

Benchmarking Unweighted Agreement Coefficients using Cumulative Membership Probabilities

| **Benchmark** | **Interpretation** | **Cohen** | **Gwet** | **Scott's** | **Krippendorff** | **Brennan** | **Percent** |
| --- | --- | --- | --- | --- | --- | --- | --- |
| **Scale** |  | **Kappa** | **AC_1_** | **Pi** | **Alpha** | **Prediger** | **Agreement** |
| 0,8 to 1 | Almost Perfect | 0,00000 | 1,00000 | 0,00000 | 0,00000 | 1,00000 | 1,00000 |
| 0,6 to 0,8 | Substantial | 0,00000 | 1,00000 | 0,00000 | 0,00000 | 1,00000 | 1,00000 |
| 0,4 to 0,6 | Moderate | 0,00000 | 1,00000 | 0,00000 | 0,00000 | 1,00000 | 1,00000 |
| 0,2 to 0,4 | Fair | 0,00000 | 1,00000 | 0,00000 | 0,00000 | 1,00000 | 1,00000 |
| 0 to 0,2 | Slight | 0,79085 | 1,00000 | 0,69565 | 0,69805 | 1,00000 | 1,00000 |
| Less than 0 | Poor | 1,00000 | 1,00000 | 1,00000 | 1,00000 | 1,00000 | 1,00000 |
