# Supplementary material for: Reliability of reporting differences in degenerative MRI findings of the lumbar spine from the supine to the upright position
Source: Skeletal Radiol. 2022 May 10;51(11):2141–54. doi: 10.1007/s00256-022-04060-2 (PMC9463326; doi:10.1007/s00256-022-04060-2)
Supplement: Supplementary file 2 — Supplementary file2 (DOCX 35 KB) [file 256_2022_4060_MOESM2_ESM.docx]

| **Inter-rater reliability of observed differences comparing** |
| --- |
| **degenerative MRI-findings in the supine and upright positions** |
| **AgreeStat 2015.6.1** |
| MODULE: Three-Rater Chance-Corrected Agreement Coefficients (Time: 17:15:47. Date: 14. juli 2020) |

| **Group:** | **MR02 Spondylolisthesis changetype** |
| --- | --- |

## DISTRIBUTION OF SUBJECTS BY RATER AND SCORE/CATEGORY (0=No change, 1=Appeared, 2=Disappeared; 3=Worsened and 4=Improved)

Category

| Raters | **0** | **1** | **2** | **3** | **4** | **Total** |
| --- | --- | --- | --- | --- | --- | --- |
| **Rater 1** | 531 | 0 | 0 | 0 | 0 | 531 |
| **Rater 2** | 530 | 0 | 0 | 1 | 0 | 531 |
| **Rater 3** | 528 | 0 | 1 | 2 | 0 | 531 |
| **Average** | 529,7 | 0 | 0,3 | 1 | 0 | 531,00 |

# UNWEIGHTED ANALYSIS

| METHOD | **Coefficient** | **Inference/Subjects** | | | **/Subjects & Raters** | | |
| --- | --- | --- | --- | --- | --- | --- | --- |
|  |  | **StdErr** | **95% C.I.** | **p-Value** | **StdErr** | **95% C.I.** | **p-Value** |
| **Conger's Kappa** | 0,24912 | 0,15280 | -0,051 to 0,549 | 1,036E-01 | 0,36597 | -0,469 to 0,967 | 4,962E-01 |
| **Gwet's AC_1_** | 0,99623 | 0,00218 | 0,992 to 1 | 0,000E+00 | 0,00308 | 0,99 to 1 | 0,000E+00 |
| **Fleiss' Kappa** | 0,24847 | 0,15309 | -0,052 to 0,549 | 1,052E-01 | 0,36683 | -0,471 to 0,968 | 4,983E-01 |
| **Krippendorff's Alpha** | 0,24894 | 0,15309 | -0,052 to 0,55 | 1,045E-01 | 0,36655 | -0,47 to 0,968 | 4,972E-01 |
| **Brenann-Prediger** | 0,99529 | 0,00271 | 0,99 to 1 | 0,000E+00 | 0,00384 | 0,988 to 1 | 0,000E+00 |
| **Percent Agreement** | 0,99623 | 0,00217 | 0,992 to 1 | 0,000E+00 | 0,00307 | 0,99 to 1 | 0,000E+00 |

## Inter-Rater Reliability

| LANDIS-KOCH INTERPRETATION OF THE AGREEMENT COEFFICIENTS (Source of Variation: Subjects Only) |
| --- |
| Benchmarking Unweighted Agreement Coefficients using Cumulative Membership Probabilities |

| **Benchmark** | **Interpretation** | **Conger'** | **Gwet** | **Fleiss'** | **Krippendorff** | **Brennan** | **Percent** |
| --- | --- | --- | --- | --- | --- | --- | --- |
| **Scale** |  | **Kappa** | **AC_1_** | **Kappa** | **Alpha** | **Prediger** | **Agreement** |
| 0,8 to 1 | Almost Perfect | 0,00016 | 1,00000 | 0,00016 | 0,00016 | 1,00000 | 1,00000 |
| 0,6 to 0,8 | Substantial | 0,01083 | 1,00000 | 0,01083 | 0,01092 | 1,00000 | 1,00000 |
| 0,4 to 0,6 | Moderate | 0,16172 | 1,00000 | 0,16114 | 0,16189 | 1,00000 | 1,00000 |
| 0,2 to 0,4 | Fair | 0,62606 | 1,00000 | 0,62422 | 0,62539 | 1,00000 | 1,00000 |
| 0 to 0,2 | Slight | 0,94848 | 1,00000 | 0,94770 | 0,94803 | 1,00000 | 1,00000 |
| Less than 0 | Poor | 1,00000 | 1,00000 | 1,00000 | 1,00000 | 1,00000 | 1,00000 |

|  |  |
| --- | --- |
| **Group:** | **MR05 Scoliosis changetype** |

**DISTRIBUTION OF SUBJECTS BY RATER AND SCORE/CATEGORY (0=No change, 1=Appeared, 2=Disappeared; 3=Worsened and 4=Improved)**

Category

| Raters | **0** | **1** | **2** | **3** | **4** | **Total** |
| --- | --- | --- | --- | --- | --- | --- |
| **Rater 1** | 175 | 1 | 0 | 1 | 0 | 177 |
| **Rater 2** | 173 | 3 | 0 | 1 | 0 | 177 |
| **Rater 3** | 174 | 3 | 0 | 0 | 0 | 177 |
| **Average** | 174 | 2,3 | 0 | 0,7 | 0 | 177,00 |

# UNWEIGHTED ANALYSIS

| METHOD | **Coefficient** | **Inference/Subjects** | | | **/Subjects & Raters** | | |
| --- | --- | --- | --- | --- | --- | --- | --- |
|  |  | **StdErr** | **95% C.I.** | **p-Value** | **StdErr** | **95% C.I.** | **p-Value** |
| **Conger's Kappa** | -0,01336 | 0,00473 | -0,023 to -0,004 | 5,275E-03 | 0,00640 | -0,026 to -0,001 | 3,757E-02 |
| **Gwet's AC_1_** | 0,96582 | 0,01123 | 0,944 to 0,988 | 0,000E+00 | 0,01304 | 0,94 to 0,991 | 0,000E+00 |
| **Fleiss' Kappa** | -0,01422 | 0,00489 | -0,024 to -0,005 | 4,077E-03 | 0,00596 | -0,026 to -0,003 | 1,751E-02 |
| **Krippendorff's Alpha** | -0,01231 | 0,00489 | -0,022 to -0,003 | 1,265E-02 | 0,00595 | -0,024 to -0,001 | 3,935E-02 |
| **Brenann-Prediger** | 0,95763 | 0,01380 | 0,93 to 0,985 | 0,000E+00 | 0,01603 | 0,926 to 0,989 | 0,000E+00 |
| **Percent Agreement** | 0,96610 | 0,01104 | 0,944 to 0,988 | 0,000E+00 | 0,01282 | 0,941 to 0,991 | 0,000E+00 |

## Inter-Rater Reliability

LANDIS-KOCH INTERPRETATION OF THE AGREEMENT COEFFICIENTS (Source of Variation: Subjects Only)

Benchmarking Unweighted Agreement Coefficients using Cumulative Membership Probabilities

| **Benchmark** | **Interpretation** | **Conger'** | **Gwet** | **Fleiss'** | **Krippendorff** | **Brennan** | **Percent** |
| --- | --- | --- | --- | --- | --- | --- | --- |
| **Scale** |  | **Kappa** | **AC_1_** | **Kappa** | **Alpha** | **Prediger** | **Agreement** |
| 0,8 to 1 | Almost Perfect | 0,00000 | 1,00000 | 0,00000 | 0,00000 | 1,00000 | 1,00000 |
| 0,6 to 0,8 | Substantial | 0,00000 | 1,00000 | 0,00000 | 0,00000 | 1,00000 | 1,00000 |
| 0,4 to 0,6 | Moderate | 0,00000 | 1,00000 | 0,00000 | 0,00000 | 1,00000 | 1,00000 |
| 0,2 to 0,4 | Fair | 0,00000 | 1,00000 | 0,00000 | 0,00000 | 1,00000 | 1,00000 |
| 0 to 0,2 | Slight | 0,00236 | 1,00000 | 0,00180 | 0,00588 | 1,00000 | 1,00000 |
| Less than 0 | Poor | 1,00000 | 1,00000 | 1,00000 | 1,00000 | 1,00000 | 1,00000 |

|  |  |
| --- | --- |
| **Group:** | **MR06 Annular fissure changetype** |

## DISTRIBUTION OF SUBJECTS BY RATER AND SCORE/CATEGORY (0=No change, 1=Appeared, 2=Disappeared; 3=Worsened and 4=Improved)

Category

| Raters | **0** | **1** | **2** | **3** | **4** | **Total** |
| --- | --- | --- | --- | --- | --- | --- |
| **Rater 1** | 172 | 0 | 1 | 1 | 3 | 177 |
| **Rater 2** | 171 | 2 | 4 | 0 | 0 | 177 |
| **Rater 3** | 168 | 3 | 5 | 1 | 0 | 177 |
| **Average** | 170,3 | 1,7 | 3,3 | 0,7 | 1 | 177,00 |

# UNWEIGHTED ANALYSIS

| METHOD | **Coefficient** | **Inference/Subjects** | | | **/Subjects & Raters** | | |
| --- | --- | --- | --- | --- | --- | --- | --- |
|  |  | **StdErr** | **95% C.I.** | **p-Value** | **StdErr** | **95% C.I.** | **p-Value** |
| **Conger's Kappa** | 0,07852 | 0,04960 | -0,019 to 0,176 | 1,152E-01 | 0,10239 | -0,123 to 0,28 | 4,436E-01 |
| **Gwet's AC_1_** | 0,93094 | 0,01647 | 0,898 to 0,963 | 0,000E+00 | 0,01784 | 0,896 to 0,966 | 0,000E+00 |
| **Fleiss' Kappa** | 0,07661 | 0,04939 | -0,021 to 0,174 | 1,227E-01 | 0,10233 | -0,125 to 0,278 | 4,546E-01 |
| **Krippendorff's Alpha** | 0,07835 | 0,04939 | -0,019 to 0,176 | 1,144E-01 | 0,10211 | -0,122 to 0,279 | 4,434E-01 |
| **Brenann-Prediger** | 0,91525 | 0,01985 | 0,876 to 0,954 | 0,000E+00 | 0,02146 | 0,873 to 0,957 | 0,000E+00 |
| **Percent Agreement** | 0,93220 | 0,01588 | 0,901 to 0,964 | 0,000E+00 | 0,01717 | 0,898 to 0,966 | 0,000E+00 |

## Inter-Rater Reliability

LANDIS-KOCH INTERPRETATION OF THE AGREEMENT COEFFICIENTS (Source of Variation: Subjects Only) Benchmarking Unweighted Agreement Coefficients using Cumulative Membership Probabilities

| **Benchmark** | **Interpretation** | **Conger'** | **Gwet** | **Fleiss'** | **Krippendorff** | **Brennan** | **Percent** |
| --- | --- | --- | --- | --- | --- | --- | --- |
| **Scale** |  | **Kappa** | **AC_1_** | **Kappa** | **Alpha** | **Prediger** | **Agreement** |
| 0,8 to 1 | Almost Perfect | 0,00000 | 1,00000 | 0,00000 | 0,00000 | 1,00000 | 1,00000 |
| 0,6 to 0,8 | Substantial | 0,00000 | 1,00000 | 0,00000 | 0,00000 | 1,00000 | 1,00000 |
| 0,4 to 0,6 | Moderate | 0,00000 | 1,00000 | 0,00000 | 0,00000 | 1,00000 | 1,00000 |
| 0,2 to 0,4 | Fair | 0,00716 | 1,00000 | 0,00624 | 0,00689 | 1,00000 | 1,00000 |
| 0 to 0,2 | Slight | 0,94329 | 1,00000 | 0,93957 | 0,94367 | 1,00000 | 1,00000 |
| Less than 0 | Poor | 1,00000 | 1,00000 | 1,00000 | 1,00000 | 1,00000 | 1,00000 |

|  |  |
| --- | --- |
| **Group:** | **MR07 Disc degeneration changetype** |

## DISTRIBUTION OF SUBJECTS BY RATER AND SCORE/CATEGORY (0=No change, 1=Appeared, 2=Disappeared; 3=Worsened and 4=Improved)

Category

| Raters | **0** | **1** | **2** | **3** | **4** | **Total** |
| --- | --- | --- | --- | --- | --- | --- |
| **Rater 1** | 175 | 0 | 0 | 0 | 2 | 177 |
| **Rater 2** | 176 | 0 | 0 | 1 | 0 | 177 |
| **Rater 3** | 175 | 0 | 0 | 2 | 0 | 177 |
| **Average** | 175,3 | 0 | 0 | 1 | 0,7 | 177,00 |

# UNWEIGHTED ANALYSIS

| METHOD | **Coefficient** | **Inference/Subjects** | | | **/Subjects & Raters** | | |
| --- | --- | --- | --- | --- | --- | --- | --- |
|  |  | **StdErr** | **95% C.I.** | **p-Value** | **StdErr** | **95% C.I.** | **p-Value** |
| **Conger's Kappa** | -0,00568 | 0,00289 | -0,011 to 0 | 5,087E-02 | 0,00363 | -0,013 to 0,001 | 1,186E-01 |
| **Gwet's AC_1_** | 0,98108 | 0,00840 | 0,964 to 0,998 | 0,000E+00 | 0,00922 | 0,963 to 0,999 | 0,000E+00 |
| **Fleiss' Kappa** | -0,00721 | 0,00324 | -0,014 to -0,001 | 2,723E-02 | 0,00348 | -0,014 to 0 | 3,900E-02 |
| **Krippendorff's Alpha** | -0,00531 | 0,00324 | -0,012 to 0,001 | 1,026E-01 | 0,00348 | -0,012 to 0,002 | 1,276E-01 |
| **Brenann-Prediger** | 0,97646 | 0,01041 | 0,956 to 0,997 | 0,000E+00 | 0,01142 | 0,954 to 0,999 | 0,000E+00 |
| **Percent Agreement** | 0,98117 | 0,00833 | 0,965 to 0,998 | 0,000E+00 | 0,00914 | 0,963 to 0,999 | 0,000E+00 |

## Inter-Rater Reliability

LANDIS-KOCH INTERPRETATION OF THE AGREEMENT COEFFICIENTS (Source of Variation: Subjects Only) Benchmarking Unweighted Agreement Coefficients using Cumulative Membership Probabilities

| **Benchmark** | **Interpretation** | **Conger'** | **Gwet** | **Fleiss'** | **Krippendorff** | **Brennan** | **Percent** |
| --- | --- | --- | --- | --- | --- | --- | --- |
| **Scale** |  | **Kappa** | **AC_1_** | **Kappa** | **Alpha** | **Prediger** | **Agreement** |
| 0,8 to 1 | Almost Perfect | 0,00000 | 1,00000 | 0,00000 | 0,00000 | 1,00000 | 1,00000 |
| 0,6 to 0,8 | Substantial | 0,00000 | 1,00000 | 0,00000 | 0,00000 | 1,00000 | 1,00000 |
| 0,4 to 0,6 | Moderate | 0,00000 | 1,00000 | 0,00000 | 0,00000 | 1,00000 | 1,00000 |
| 0,2 to 0,4 | Fair | 0,00000 | 1,00000 | 0,00000 | 0,00000 | 1,00000 | 1,00000 |
| 0 to 0,2 | Slight | 0,02465 | 1,00000 | 0,01298 | 0,05042 | 1,00000 | 1,00000 |
| Less than 0 | Poor | 1,00000 | 1,00000 | 1,00000 | 1,00000 | 1,00000 | 1,00000 |

|  |  |
| --- | --- |
| **Group:** | **MR08 Disc contour changetype** |

## DISTRIBUTION OF SUBJECTS BY RATER AND SCORE/CATEGORY (0=No change, 1=Appeared, 2=Disappeared; 3=Worsened and 4=Improved)

Category

| Raters | **0** | **1** | **2** | **3** | **4** | **Total** |
| --- | --- | --- | --- | --- | --- | --- |
| **Rater 1** | 353 | 1 | 0 | 0 | 0 | 354 |
| **Rater 2** | 339 | 5 | 1 | 9 | 0 | 354 |
| **Rater 3** | 328 | 5 | 0 | 20 | 1 | 354 |
| **Average** | 340 | 3,7 | 0,3 | 9,7 | 0,3 | 354,00 |

# UNWEIGHTED ANALYSIS

| METHOD | **Coefficient** | **Inference/Subjects** | | | **/Subjects & Raters** | | |
| --- | --- | --- | --- | --- | --- | --- | --- |
|  |  | **StdErr** | **95% C.I.** | **p-Value** | **StdErr** | **95% C.I.** | **p-Value** |
| **Conger's Kappa** | 0,11165 | 0,03969 | 0,034 to 0,19 | 5,184E-03 | 0,13852 | -0,16 to 0,384 | 4,205E-01 |
| **Gwet's AC_1_** | 0,92992 | 0,01159 | 0,907 to 0,953 | 0,000E+00 | 0,02849 | 0,874 to 0,986 | 0,000E+00 |
| **Fleiss' Kappa** | 0,10354 | 0,04013 | 0,025 to 0,182 | 1,028E-02 | 0,14827 | -0,188 to 0,395 | 4,852E-01 |
| **Krippendorff's Alpha** | 0,10438 | 0,04013 | 0,025 to 0,183 | 9,680E-03 | 0,14807 | -0,186 to 0,395 | 4,811E-01 |
| **Brenann-Prediger** | 0,91408 | 0,01394 | 0,887 to 0,942 | 0,000E+00 | 0,03412 | 0,847 to 0,981 | 0,000E+00 |
| **Percent Agreement** | 0,93126 | 0,01116 | 0,909 to 0,953 | 0,000E+00 | 0,02730 | 0,878 to 0,985 | 0,000E+00 |

## Inter-Rater Reliability

LANDIS-KOCH INTERPRETATION OF THE AGREEMENT COEFFICIENTS (Source of Variation: Subjects Only) Benchmarking Unweighted Agreement Coefficients using Cumulative Membership Probabilities

| **Benchmark** | **Interpretation** | **Conger'** | **Gwet** | **Fleiss'** | **Krippendorff** | **Brennan** | **Percent** |
| --- | --- | --- | --- | --- | --- | --- | --- |
| **Scale** |  | **Kappa** | **AC_1_** | **Kappa** | **Alpha** | **Prediger** | **Agreement** |
| 0,8 to 1 | Almost Perfect | 0,00000 | 1,00000 | 0,00000 | 0,00000 | 1,00000 | 1,00000 |
| 0,6 to 0,8 | Substantial | 0,00000 | 1,00000 | 0,00000 | 0,00000 | 1,00000 | 1,00000 |
| 0,4 to 0,6 | Moderate | 0,00000 | 1,00000 | 0,00000 | 0,00000 | 1,00000 | 1,00000 |
| 0,2 to 0,4 | Fair | 0,01302 | 1,00000 | 0,00811 | 0,00859 | 1,00000 | 1,00000 |
| 0 to 0,2 | Slight | 0,99755 | 1,00000 | 0,99506 | 0,99536 | 1,00000 | 1,00000 |
| Less than 0 | Poor | 1,00000 | 1,00000 | 1,00000 | 1,00000 | 1,00000 | 1,00000 |

|  |  |
| --- | --- |
| **Group:** | **MR09 Nerve compromise changetype** |

## DISTRIBUTION OF SUBJECTS BY RATER AND SCORE/CATEGORY (0=No change, 1=Appeared, 2=Disappeared; 3=Worsened and 4=Improved)

Category

| Raters | **0** | **1** | **2** | **3** | **4** | **Total** |
| --- | --- | --- | --- | --- | --- | --- |
| **Rater 1** | 174 | 0 | 0 | 2 | 1 | 177 |
| **Rater 2** | 168 | 4 | 0 | 5 | 0 | 177 |
| **Rater 3** | 169 | 6 | 0 | 2 | 0 | 177 |
| **Average** | 170,3 | 3,3 | 0 | 3 | 0,3 | 177,00 |

# UNWEIGHTED ANALYSIS

| METHOD | **Coefficient** | **Inference/Subjects** | | | **/Subjects & Raters** | | |
| --- | --- | --- | --- | --- | --- | --- | --- |
|  |  | **StdErr** | **95% C.I.** | **p-Value** | **StdErr** | **95% C.I.** | **p-Value** |
| **Conger's Kappa** | 0,05211 | 0,04835 | -0,043 to 0,148 | 2,827E-01 | 0,08208 | -0,109 to 0,214 | 5,260E-01 |
| **Gwet's AC_1_** | 0,92902 | 0,01630 | 0,897 to 0,961 | 0,000E+00 | 0,02362 | 0,883 to 0,975 | 0,000E+00 |
| **Fleiss' Kappa** | 0,04894 | 0,04838 | -0,047 to 0,144 | 3,131E-01 | 0,08416 | -0,117 to 0,214 | 5,613E-01 |
| **Krippendorff's Alpha** | 0,05073 | 0,04838 | -0,045 to 0,146 | 2,958E-01 | 0,08400 | -0,114 to 0,216 | 5,463E-01 |
| **Brenann-Prediger** | 0,91290 | 0,01965 | 0,874 to 0,952 | 0,000E+00 | 0,02841 | 0,857 to 0,969 | 0,000E+00 |

## Inter-Rater Reliability

| **Percent Agreement** | 0,93032 | 0,01572 | 0,899 to 0,961 | 0,000E+00 | 0,02273 | 0,886 to 0,975 | 0,000E+00 |
| --- | --- | --- | --- | --- | --- | --- | --- |

LANDIS-KOCH INTERPRETATION OF THE AGREEMENT COEFFICIENTS (Source of Variation: Subjects Only) Benchmarking Unweighted Agreement Coefficients using Cumulative Membership Probabilities

| **Benchmark** | **Interpretation** | **Conger'** | **Gwet** | **Fleiss'** | **Krippendorff** | **Brennan** | **Percent** |
| --- | --- | --- | --- | --- | --- | --- | --- |
| **Scale** |  | **Kappa** | **AC_1_** | **Kappa** | **Alpha** | **Prediger** | **Agreement** |
| 0,8 to 1 | Almost Perfect | 0,00000 | 1,00000 | 0,00000 | 0,00000 | 1,00000 | 1,00000 |
| 0,6 to 0,8 | Substantial | 0,00000 | 1,00000 | 0,00000 | 0,00000 | 1,00000 | 1,00000 |
| 0,4 to 0,6 | Moderate | 0,00000 | 1,00000 | 0,00000 | 0,00000 | 1,00000 | 1,00000 |
| 0,2 to 0,4 | Fair | 0,00111 | 1,00000 | 0,00090 | 0,00102 | 1,00000 | 1,00000 |
| 0 to 0,2 | Slight | 0,85939 | 1,00000 | 0,84415 | 0,85283 | 1,00000 | 1,00000 |
| Less than 0 | Poor | 1,00000 | 1,00000 | 1,00000 | 1,00000 | 1,00000 | 1,00000 |

|  |  |
| --- | --- |
| **Group:** | **MR12 Spinal stenosis changetype** |

## DISTRIBUTION OF SUBJECTS BY RATER AND SCORE/CATEGORY (0=No change, 1=Appeared, 2=Disappeared; 3=Worsened and 4=Improved)

Category

| Raters | **0** | **1** | **2** | **3** | **4** | **Total** |
| --- | --- | --- | --- | --- | --- | --- |
| **Rater 1** | 880 | 0 | 0 | 1 | 4 | 885 |
| **Rater 2** | 853 | 12 | 0 | 19 | 1 | 885 |
| **Rater 3** | 858 | 16 | 0 | 9 | 2 | 885 |
| **Average** | 863,7 | 9,3 | 0 | 9,7 | 2,3 | 885,00 |

# UNWEIGHTED ANALYSIS

| METHOD | **Coefficient** | **Inference/Subjects** | | | **/Subjects & Raters** | | |
| --- | --- | --- | --- | --- | --- | --- | --- |
|  |  | **StdErr** | **95% C.I.** | **p-Value** | **StdErr** | **95% C.I.** | **p-Value** |
| **Conger's Kappa** | 0,12067 | 0,03727 | 0,048 to 0,194 | 1,250E-03 | 0,10168 | -0,079 to 0,32 | 2,355E-01 |
| **Gwet's AC_1_** | 0,95769 | 0,00576 | 0,946 to 0,969 | 0,000E+00 | 0,01404 | 0,93 to 0,985 | 0,000E+00 |
| **Fleiss' Kappa** | 0,11783 | 0,03752 | 0,044 to 0,191 | 1,740E-03 | 0,10626 | -0,091 to 0,326 | 2,676E-01 |
| **Krippendorff's Alpha** | 0,11817 | 0,03752 | 0,045 to 0,192 | 1,689E-03 | 0,10620 | -0,09 to 0,326 | 2,660E-01 |

## Inter-Rater Reliability

| **Brenann-Prediger** | 0,94774 | 0,00704 | 0,934 to 0,962 | 0,000E+00 | 0,01708 | 0,914 to 0,981 | 0,000E+00 |
| --- | --- | --- | --- | --- | --- | --- | --- |
| **Percent Agreement** | 0,95819 | 0,00563 | 0,947 to 0,969 | 0,000E+00 | 0,01366 | 0,931 to 0,985 | 0,000E+00 |

LANDIS-KOCH INTERPRETATION OF THE AGREEMENT COEFFICIENTS (Source of Variation: Subjects Only) Benchmarking Unweighted Agreement Coefficients using Cumulative Membership Probabilities

| **Benchmark** | **Interpretation** | **Conger'** | **Gwet** | **Fleiss'** | **Krippendorff** | **Brennan** | **Percent** |
| --- | --- | --- | --- | --- | --- | --- | --- |
| **Scale** |  | **Kappa** | **AC_1_** | **Kappa** | **Alpha** | **Prediger** | **Agreement** |
| 0,8 to 1 | Almost Perfect | 0,00000 | 1,00000 | 0,00000 | 0,00000 | 1,00000 | 1,00000 |
| 0,6 to 0,8 | Substantial | 0,00000 | 1,00000 | 0,00000 | 0,00000 | 1,00000 | 1,00000 |
| 0,4 to 0,6 | Moderate | 0,00000 | 1,00000 | 0,00000 | 0,00000 | 1,00000 | 1,00000 |
| 0,2 to 0,4 | Fair | 0,01665 | 1,00000 | 0,01426 | 0,01458 | 1,00000 | 1,00000 |
| 0 to 0,2 | Slight | 0,99940 | 1,00000 | 0,99916 | 0,99918 | 1,00000 | 1,00000 |
| Less than 0 | Poor | 1,00000 | 1,00000 | 1,00000 | 1,00000 | 1,00000 | 1,00000 |

|  |  |
| --- | --- |
| **Group:** | **MR14 Facet degeneration changetype** |

## DISTRIBUTION OF SUBJECTS BY RATER AND SCORE/CATEGORY (0=No change, 1=Appeared, 2=Disappeared; 3=Worsened and 4=Improved)

Category

| Raters | **0** | **1** | **2** | **3** | **4** | **Total** |
| --- | --- | --- | --- | --- | --- | --- |
| **Rater 1** | 531 | 0 | 0 | 0 | 0 | 531 |
| **Rater 2** | 528 | 3 | 0 | 0 | 0 | 531 |
| **Rater 3** | 529 | 2 | 0 | 0 | 0 | 531 |
| **Average** | 529,3 | 1,7 | 0 | 0 | 0 | 531,00 |

# UNWEIGHTED ANALYSIS

| METHOD | **Coefficient** | **Inference/Subjects** | | | **/Subjects & Raters** | | |
| --- | --- | --- | --- | --- | --- | --- | --- |
|  |  | **StdErr** | **95% C.I.** | **p-Value** | **StdErr** | **95% C.I.** | **p-Value** |
| **Conger's Kappa** | -0,00227 | 0,00109 | -0,004 to 0 | 3,875E-02 | 0,00322 | -0,009 to 0,004 | 4,817E-01 |
| **Gwet's AC_1_** | 0,99371 | 0,00281 | 0,988 to 0,999 | 0,000E+00 | 0,00436 | 0,985 to 1 | 0,000E+00 |
| **Fleiss' Kappa** | -0,00315 | 0,00141 | -0,006 to 0 | 2,567E-02 | 0,00219 | -0,007 to 0,001 | 1,500E-01 |

## Inter-Rater Reliability

| **Krippendorff's Alpha** | -0,00252 | 0,00141 | -0,005 to 0 | 7,402E-02 | 0,00218 | -0,007 to 0,002 | 2,491E-01 |
| --- | --- | --- | --- | --- | --- | --- | --- |
| **Brenann-Prediger** | 0,99215 | 0,00350 | 0,985 to 0,999 | 0,000E+00 | 0,00543 | 0,982 to 1 | 0,000E+00 |
| **Percent Agreement** | 0,99372 | 0,00280 | 0,988 to 0,999 | 0,000E+00 | 0,00434 | 0,985 to 1 | 0,000E+00 |

LANDIS-KOCH INTERPRETATION OF THE AGREEMENT COEFFICIENTS (Source of Variation: Subjects Only) Benchmarking Unweighted Agreement Coefficients using Cumulative Membership Probabilities

| **Benchmark** | **Interpretation** | **Conger'** | **Gwet** | **Fleiss'** | **Krippendorff** | **Brennan** | **Percent** |
| --- | --- | --- | --- | --- | --- | --- | --- |
| **Scale** |  | **Kappa** | **AC_1_** | **Kappa** | **Alpha** | **Prediger** | **Agreement** |
| 0,8 to 1 | Almost Perfect | 0,00000 | 1,00000 | 0,00000 | 0,00000 | 1,00000 | 1,00000 |
| 0,6 to 0,8 | Substantial | 0,00000 | 1,00000 | 0,00000 | 0,00000 | 1,00000 | 1,00000 |
| 0,4 to 0,6 | Moderate | 0,00000 | 1,00000 | 0,00000 | 0,00000 | 1,00000 | 1,00000 |
| 0,2 to 0,4 | Fair | 0,00000 | 1,00000 | 0,00000 | 0,00000 | 1,00000 | 1,00000 |
| 0 to 0,2 | Slight | 0,01914 | 1,00000 | 0,01263 | 0,03673 | 1,00000 | 1,00000 |
| Less than 0 | Poor | 1,00000 | 1,00000 | 1,00000 | 1,00000 | 1,00000 | 1,00000 |
