# Supplementary material for: Reliability of reporting differences in degenerative MRI findings of the lumbar spine from the supine to the upright position
Source: Skeletal Radiol. 2022 May 10;51(11):2141–54. doi: 10.1007/s00256-022-04060-2 (PMC9463326; doi:10.1007/s00256-022-04060-2)
Supplement: Supplementary file 3 — Supplementary file3 (DOCX 38 KB) [file 256_2022_4060_MOESM3_ESM.docx]

| **Inter-rater reliability of observed differences comparing** |
| --- |
| **degenerative MRI-findings in the supine and upright positions** |
| **AgreeStat 2015.6.1** |
| MODULE: Two-Rater Chance-Corrected Agreement Coefficients (Time: 16:52:24. Date: 14. juli 2020) |

**Group: Spondylolisthesis MR02changetype**

Rater 1

|  | **0** | **1** | **2** | **3** | **4** | **Missing** | **Total** |  |
| --- | --- | --- | --- | --- | --- | --- | --- | --- |
| **0** | 528 | 0 | 1 | 2 | 0 | 0 | 531 | [100%] |
| **1** | 0 | 0 | 0 | 0 | 0 | 0 | 0 | [0%] |
| **2** | 0 | 0 | 0 | 0 | 0 | 0 | 0 | [0%] |
| **3** | 0 | 0 | 0 | 0 | 0 | 0 | 0 | [0%] |
| **4** | 0 | 0 | 0 | 0 | 0 | 0 | 0 | [0%] |
| **Missing** | 0 | 0 | 0 | 0 | 0 | 0 | 0 | [0%] |
| **Total** | 528 | 0 | 1 | 2 | 0 | 0 | 531 | [100%] |
| [99,4%] | | [0%] | [0,2%] | [0,4%] | [0%] | [0%] | [100%] |  |

DISTRIBUTION OF SUBJECTS BY RATER AND CATEGORY (0=No change, 1=Appeared, 2=Disappeared; 3=Worsened and 4=Improved) Rater 3

## INTER-RATER RELIABILITY COEFFICIENTS AND ASSOCIATED PRECISION MEASURES

Unweighted Agreement Coefficients

| METHOD | **Coeff.** | **StdErr** | **95% C.I.** | **p-Value** |
| --- | --- | --- | --- | --- |
| **Cohen's Kappa** | 0,00000 | 1,13024E-14 | 0 to 0 | 8,268E-02 |
| **Gwet's AC_1_** | 0,99434 | 0,003264892 | 0,988 to 1 | 0,000E+00 |
| **Scott's Pi** | -0,00220 | 0,001297528 | -0,005 to 0 | 9,028E-02 |
| **Krippendorff's Alpha** | -0,00126 | 0,001297528 | -0,004 to 0,001 | 3,326E-01 |
| **Brenann-Prediger** | 0,99294 | 0,004069632 | 0,985 to 1 | 0,000E+00 |
| **Percent Agreement** | 0,99435 | 0,003255706 | 0,988 to 1 | 0,000E+00 |

# LANDIS-KOCH INTERPRETATION OF THE AGREEMENT COEFFICIENTS

Benchmarking Unweighted Agreement Coefficients using Cumulative Membership Probabilities

| **Benchmark** | **Interpretation** | **Cohen** | **Gwet** | **Scott's** | **Krippendorff** | **Brennan** | **Percent** |
| --- | --- | --- | --- | --- | --- | --- | --- |
| **Scale** |  | **Kappa** | **AC_1_** | **Pi** | **Alpha** | **Prediger** | **Agreement** |
| 0,8 to 1 | Almost Perfect | 0,00000 | 1,00000 | 0,00000 | 0,00000 | 1,00000 | 1,00000 |
| 0,6 to 0,8 | Substantial | 0,00000 | 1,00000 | 0,00000 | 0,00000 | 1,00000 | 1,00000 |
| 0,4 to 0,6 | Moderate | 0,00000 | 1,00000 | 0,00000 | 0,00000 | 1,00000 | 1,00000 |
| 0,2 to 0,4 | Fair | 0,00000 | 1,00000 | 0,00000 | 0,00000 | 1,00000 | 1,00000 |
| 0 to 0,2 | Slight | 0,04105 | 1,00000 | 0,04484 | 0,16609 | 1,00000 | 1,00000 |
| Less than 0 | Poor | 1,00000 | 1,00000 | 1,00000 | 1,00000 | 1,00000 | 1,00000 |

**Group: Scoliosis MR05changetype**

Rater 1

|  | **0** | **1** | **2** | **3** | **4** | **Missing** | **Total** |  |
| --- | --- | --- | --- | --- | --- | --- | --- | --- |
| **0** | 172 | 3 | 0 | 0 | 0 | 0 | 175 | [98,9%] |
| **1** | 1 | 0 | 0 | 0 | 0 | 0 | 1 | [0,6%] |
| **2** | 0 | 0 | 0 | 0 | 0 | 0 | 0 | [0%] |
| **3** | 1 | 0 | 0 | 0 | 0 | 0 | 1 | [0,6%] |
| **4** | 0 | 0 | 0 | 0 | 0 | 0 | 0 | [0%] |
| **Missing** | 0 | 0 | 0 | 0 | 0 | 0 | 0 | [0%] |
| **Total** | 174 | 3 | 0 | 0 | 0 | 0 | 177 | [100%] |
| [98,3%] | | [1,7%] | [0%] | [0%] | [0%] | [0%] | [100%] |  |

DISTRIBUTION OF SUBJECTS BY RATER AND CATEGORY (0=No change, 1=Appeared, 2=Disappeared; 3=Worsened and 4=Improved) Rater 3

## INTER-RATER RELIABILITY COEFFICIENTS AND ASSOCIATED PRECISION MEASURES

Unweighted Agreement Coefficients

| METHOD | **Coeff.** | **StdErr** | **95% C.I.** | **p-Value** |
| --- | --- | --- | --- | --- |
| **Cohen's Kappa** | -0,01027 | 0,005539678 | -0,021 to 0,001 | 6,532E-02 |

| **Gwet's AC_1_** | 0,97155 | 0,012663851 | 0,947 to 0,997 | 3,322E-137 |
| --- | --- | --- | --- | --- |
| **Scott's Pi** | -0,01201 | 0,005593087 | -0,023 to -0,001 | 3,318E-02 |
| **Krippendorff's Alpha** | -0,00915 | 0,005593087 | -0,02 to 0,002 | 1,037E-01 |
| **Brenann-Prediger** | 0,96469 | 0,015610961 | 0,934 to 0,995 | 2,902E-121 |
| **Percent Agreement** | 0,97175 | 0,012488769 | 0,947 to 0,996 | 2,968E-138 |

# LANDIS-KOCH INTERPRETATION OF THE AGREEMENT COEFFICIENTS

Benchmarking Unweighted Agreement Coefficients using Cumulative Membership Probabilities

| **Benchmark** | **Interpretation** | **Cohen** | **Gwet** | **Scott's** | **Krippendorff** | **Brennan** | **Percent** |
| --- | --- | --- | --- | --- | --- | --- | --- |
| **Scale** |  | **Kappa** | **AC_1_** | **Pi** | **Alpha** | **Prediger** | **Agreement** |
| 0,8 to 1 | Almost Perfect | 0,00000 | 1,00000 | 0,00000 | 0,00000 | 1,00000 | 1,00000 |
| 0,6 to 0,8 | Substantial | 0,00000 | 1,00000 | 0,00000 | 0,00000 | 1,00000 | 1,00000 |
| 0,4 to 0,6 | Moderate | 0,00000 | 1,00000 | 0,00000 | 0,00000 | 1,00000 | 1,00000 |
| 0,2 to 0,4 | Fair | 0,00000 | 1,00000 | 0,00000 | 0,00000 | 1,00000 | 1,00000 |
| 0 to 0,2 | Slight | 0,03183 | 1,00000 | 0,01591 | 0,05096 | 1,00000 | 1,00000 |
| Less than 0 | Poor | 1,00000 | 1,00000 | 1,00000 | 1,00000 | 1,00000 | 1,00000 |

**Group: Annular fissure MR06changetype**

Rater 1

|  | **0** | **1** | **2** | **3** | **4** | **Missing** | **Total** |  |
| --- | --- | --- | --- | --- | --- | --- | --- | --- |
| **0** | 165 | 3 | 3 | 1 | 0 | 0 | 172 | [97,2%] |
| **1** | 0 | 0 | 0 | 0 | 0 | 0 | 0 | [0%] |
| **2** | 1 | 0 | 0 | 0 | 0 | 0 | 1 | [0,6%] |
| **3** | 1 | 0 | 0 | 0 | 0 | 0 | 1 | [0,6%] |
| **4** | 1 | 0 | 2 | 0 | 0 | 0 | 3 | [1,7%] |
| **Missing** | 0 | 0 | 0 | 0 | 0 | 0 | 0 | [0%] |
| **Total** | 168 | 3 | 5 | 1 | 0 | 0 | 177 | [100%] |
| [94,9%] | | [1,7%] | [2,8%] | [0,6%] | [0%] | [0%] | [100%] |  |

DISTRIBUTION OF SUBJECTS BY RATER AND CATEGORY (0=No change, 1=Appeared, 2=Disappeared; 3=Worsened and 4=Improved) Rater 3

## INTER-RATER RELIABILITY COEFFICIENTS AND ASSOCIATED PRECISION MEASURES

Unweighted Agreement Coefficients

| METHOD | **Coeff.** | **StdErr** | **95% C.I.** | **p-Value** |
| --- | --- | --- | --- | --- |
| **Cohen's Kappa** | 0,12485 | 0,079220756 | -0,031 to 0,281 | 1,168E-01 |
| **Gwet's AC_1_** | 0,93087 | 0,019692003 | 0,892 to 0,97 | 5,929E-102 |
| **Scott's Pi** | 0,12031 | 0,07927781 | -0,036 to 0,277 | 1,309E-01 |
| **Krippendorff's Alpha** | 0,12280 | 0,07927781 | -0,034 to 0,279 | 1,232E-01 |
| **Brenann-Prediger** | 0,91525 | 0,023687161 | 0,869 to 0,962 | 6,786E-88 |
| **Percent Agreement** | 0,93220 | 0,018949729 | 0,895 to 0,97 | 8,708E-105 |

# LANDIS-KOCH INTERPRETATION OF THE AGREEMENT COEFFICIENTS

Benchmarking Unweighted Agreement Coefficients using Cumulative Membership Probabilities

| **Benchmark** | **Interpretation** | **Cohen** | **Gwet** | **Scott's** | **Krippendorff** | **Brennan** | **Percent** |
| --- | --- | --- | --- | --- | --- | --- | --- |
| **Scale** |  | **Kappa** | **AC_1_** | **Pi** | **Alpha** | **Prediger** | **Agreement** |
| 0,8 to 1 | Almost Perfect | 0,00000 | 1,00000 | 0,00000 | 0,00000 | 1,00000 | 1,00000 |
| 0,6 to 0,8 | Substantial | 0,00000 | 1,00000 | 0,00000 | 0,00000 | 1,00000 | 1,00000 |
| 0,4 to 0,6 | Moderate | 0,00026 | 1,00000 | 0,00021 | 0,00024 | 1,00000 | 1,00000 |
| 0,2 to 0,4 | Fair | 0,17139 | 1,00000 | 0,15741 | 0,16508 | 1,00000 | 1,00000 |
| 0 to 0,2 | Slight | 0,94248 | 1,00000 | 0,93545 | 0,93931 | 1,00000 | 1,00000 |
| Less than 0 | Poor | 1,00000 | 1,00000 | 1,00000 | 1,00000 | 1,00000 | 1,00000 |

**Group: Disc degeneration MR07changetype**

Rater 1

DISTRIBUTION OF SUBJECTS BY RATER AND CATEGORY (0=No change, 1=Appeared, 2=Disappeared; 3=Worsened and 4=Improved) Rater 3

[98,9%]

|  | **0** | **1** | **2** | **3** | **4** | **Missing** | **Total** |
| --- | --- | --- | --- | --- | --- | --- | --- |
| **0** | 173 | 0 | 0 | 2 | 0 | 0 | 175 |
| **1** | 0 | 0 | 0 | 0 | 0 | 0 | 0 |
| **2** | 0 | 0 | 0 | 0 | 0 | 0 | 0 |
| **3** | 0 | 0 | 0 | 0 | 0 | 0 | 0 |

[0%]

[0%]

[0%]

| **4** | 2 | 0 | 0 | 0 | 0 | 0 | 2 | [1,1%] |
| --- | --- | --- | --- | --- | --- | --- | --- | --- |
| **Missing** | 0 | 0 | 0 | 0 | 0 | 0 | 0 | [0%] |
| **Total** | 175 | 0 | 0 | 2 | 0 | 0 | 177 | [100%] |
|  | [98,9%] | [0%] | [0%] | [1,1%] | [0%] | [0%] | [100%] |  |

## INTER-RATER RELIABILITY COEFFICIENTS AND ASSOCIATED PRECISION MEASURES

Unweighted Agreement Coefficients

| METHOD | **Coeff.** | **StdErr** | **95% C.I.** | **p-Value** |
| --- | --- | --- | --- | --- |
| **Cohen's Kappa** | -0,00568 | 0,002832596 | -0,011 to 0 | 4,640E-02 |
| **Gwet's AC_1_** | 0,97727 | 0,011328751 | 0,955 to 1 | 6,145E-146 |
| **Scott's Pi** | -0,00855 | 0,004273139 | -0,017 to 0 | 4,702E-02 |
| **Krippendorff's Alpha** | -0,00570 | 0,004273139 | -0,014 to 0,003 | 1,841E-01 |
| **Brenann-Prediger** | 0,97175 | 0,014003399 | 0,944 to 0,999 | 8,849E-130 |
| **Percent Agreement** | 0,97740 | 0,011202719 | 0,955 to 1 | 8,772E-147 |

# LANDIS-KOCH INTERPRETATION OF THE AGREEMENT COEFFICIENTS

Benchmarking Unweighted Agreement Coefficients using Cumulative Membership Probabilities

| **Benchmark** | **Interpretation** | **Cohen** | **Gwet** | **Scott's** | **Krippendorff** | **Brennan** | **Percent** |
| --- | --- | --- | --- | --- | --- | --- | --- |
| **Scale** |  | **Kappa** | **AC_1_** | **Pi** | **Alpha** | **Prediger** | **Agreement** |
| 0,8 to 1 | Almost Perfect | 0,00000 | 1,00000 | 0,00000 | 0,00000 | 1,00000 | 1,00000 |
| 0,6 to 0,8 | Substantial | 0,00000 | 1,00000 | 0,00000 | 0,00000 | 1,00000 | 1,00000 |
| 0,4 to 0,6 | Moderate | 0,00000 | 1,00000 | 0,00000 | 0,00000 | 1,00000 | 1,00000 |
| 0,2 to 0,4 | Fair | 0,00000 | 1,00000 | 0,00000 | 0,00000 | 1,00000 | 1,00000 |
| 0 to 0,2 | Slight | 0,02244 | 1,00000 | 0,02274 | 0,09119 | 1,00000 | 1,00000 |
| Less than 0 | Poor | 1,00000 | 1,00000 | 1,00000 | 1,00000 | 1,00000 | 1,00000 |

**Group: Disc contour MR08changetype**

| DISTRIBUTION OF SUBJECTS BY RATER AND CATEGORY (0=No change, 1=Appeared, 2=Disappeared; 3=Worsened and 4=Improved) |
| --- |
| Rater 3 |

Rater 1

|  | **0** | **1** | **2** | **3** | **4** | **Missing** | **Total** |  |
| --- | --- | --- | --- | --- | --- | --- | --- | --- |
| **0** | 328 | 5 | 0 | 19 | 1 | 0 | 353 | [99,7%] |
| **1** | 0 | 0 | 0 | 1 | 0 | 0 | 1 | [0,3%] |
| **2** | 0 | 0 | 0 | 0 | 0 | 0 | 0 | [0%] |
| **3** | 0 | 0 | 0 | 0 | 0 | 0 | 0 | [0%] |
| **4** | 0 | 0 | 0 | 0 | 0 | 0 | 0 | [0%] |
| **Missing** | 0 | 0 | 0 | 0 | 0 | 0 | 0 | [0%] |
| **Total** | 328 | 5 | 0 | 20 | 1 | 0 | 354 | [100%] |
|  | [92,7%] | [1,4%] | [0%] | [5,6%] | [0,3%] | [0%] | [100%] |  |

## INTER-RATER RELIABILITY COEFFICIENTS AND ASSOCIATED PRECISION MEASURES

Unweighted Agreement Coefficients

| METHOD | **Coeff.** | **StdErr** | **95% C.I.** | **p-Value** |
| --- | --- | --- | --- | --- |
| **Cohen's Kappa** | 0,03390 | 0,032189625 | -0,029 to 0,097 | 2,929E-01 |
| **Gwet's AC_1_** | 0,92517 | 0,014404236 | 0,897 to 0,953 | 8,036E-197 |
| **Scott's Pi** | 0,00674 | 0,035867634 | -0,064 to 0,077 | 8,509E-01 |
| **Krippendorff's Alpha** | 0,00815 | 0,035867634 | -0,062 to 0,079 | 8,204E-01 |
| **Brenann-Prediger** | 0,90819 | 0,017355734 | 0,874 to 0,942 | 2,128E-168 |
| **Percent Agreement** | 0,92655 | 0,013884587 | 0,899 to 0,954 | 3,076E-202 |

# LANDIS-KOCH INTERPRETATION OF THE AGREEMENT COEFFICIENTS

Benchmarking Unweighted Agreement Coefficients using Cumulative Membership Probabilities

| **Benchmark** | **Interpretation** | **Cohen** | **Gwet** | **Scott's** | **Krippendorff** | **Brennan** | **Percent** |
| --- | --- | --- | --- | --- | --- | --- | --- |
| **Scale** |  | **Kappa** | **AC_1_** | **Pi** | **Alpha** | **Prediger** | **Agreement** |
| 0,8 to 1 | Almost Perfect | 0,00000 | 1,00000 | 0,00000 | 0,00000 | 1,00000 | 1,00000 |
| 0,6 to 0,8 | Substantial | 0,00000 | 1,00000 | 0,00000 | 0,00000 | 1,00000 | 1,00000 |
| 0,4 to 0,6 | Moderate | 0,00000 | 1,00000 | 0,00000 | 0,00000 | 1,00000 | 1,00000 |
| 0,2 to 0,4 | Fair | 0,00000 | 1,00000 | 0,00000 | 0,00000 | 1,00000 | 1,00000 |
| 0 to 0,2 | Slight | 0,85389 | 1,00000 | 0,57458 | 0,58985 | 1,00000 | 1,00000 |
| Less than 0 | Poor | 1,00000 | 1,00000 | 1,00000 | 1,00000 | 1,00000 | 1,00000 |

**Group: Nerve compromise MR09changetype**

Rater 1

|  | **0** | **1** | **2** | **3** | **4** | **Missing** | **Total** |  |
| --- | --- | --- | --- | --- | --- | --- | --- | --- |
| **0** | 167 | 5 | 0 | 2 | 0 | 0 | 174 | [98,3%] |
| **1** | 0 | 0 | 0 | 0 | 0 | 0 | 0 | [0%] |
| **2** | 0 | 0 | 0 | 0 | 0 | 0 | 0 | [0%] |
| **3** | 1 | 1 | 0 | 0 | 0 | 0 | 2 | [1,1%] |
| **4** | 1 | 0 | 0 | 0 | 0 | 0 | 1 | [0,6%] |
| **Missing** | 0 | 0 | 0 | 0 | 0 | 0 | 0 | [0%] |
| **Total** | 169 | 6 | 0 | 2 | 0 | 0 | 177 | [100%] |
| [95,5%] | | [3,4%] | [0%] | [1,1%] | [0%] | [0%] | [100%] |  |

DISTRIBUTION OF SUBJECTS BY RATER AND CATEGORY (0=No change, 1=Appeared, 2=Disappeared; 3=Worsened and 4=Improved) Rater 3

## INTER-RATER RELIABILITY COEFFICIENTS AND ASSOCIATED PRECISION MEASURES

Unweighted Agreement Coefficients

| METHOD | **Coeff.** | **StdErr** | **95% C.I.** | **p-Value** |
| --- | --- | --- | --- | --- |
| **Cohen's Kappa** | 0,07764 | 0,07736911 | -0,075 to 0,23 | 3,170E-01 |
| **Gwet's AC_1_** | 0,94263 | 0,017938097 | 0,907 to 0,978 | 1,645E-109 |
| **Scott's Pi** | 0,07013 | 0,078772488 | -0,085 to 0,226 | 3,745E-01 |
| **Krippendorff's Alpha** | 0,07276 | 0,078772488 | -0,083 to 0,228 | 3,569E-01 |
| **Brenann-Prediger** | 0,92938 | 0,021753976 | 0,886 to 0,972 | 7,764E-95 |
| **Percent Agreement** | 0,94350 | 0,017403181 | 0,909 to 0,978 | 9,320E-112 |

# LANDIS-KOCH INTERPRETATION OF THE AGREEMENT COEFFICIENTS

Benchmarking Unweighted Agreement Coefficients using Cumulative Membership Probabilities

| **Benchmark** | **Interpretation** | **Cohen** | **Gwet** | **Scott's** | **Krippendorff** | **Brennan** | **Percent** |
| --- | --- | --- | --- | --- | --- | --- | --- |
| **Scale** |  | **Kappa** | **AC_1_** | **Pi** | **Alpha** | **Prediger** | **Agreement** |
| 0,8 to 1 | Almost Perfect | 0,00000 | 1,00000 | 0,00000 | 0,00000 | 1,00000 | 1,00000 |
| 0,6 to 0,8 | Substantial | 0,00000 | 1,00000 | 0,00000 | 0,00000 | 1,00000 | 1,00000 |

| 0,4 to 0,6 | Moderate | 0,00002 | 1,00000 | 0,00001 | 0,00002 | 1,00000 | 1,00000 |
| --- | --- | --- | --- | --- | --- | --- | --- |
| 0,2 to 0,4 | Fair | 0,05689 | 1,00000 | 0,04961 | 0,05313 | 1,00000 | 1,00000 |
| 0 to 0,2 | Slight | 0,84220 | 1,00000 | 0,81336 | 0,82217 | 1,00000 | 1,00000 |
| Less than 0 | Poor | 1,00000 | 1,00000 | 1,00000 | 1,00000 | 1,00000 | 1,00000 |

**Group: Spinal stenosis MR12changetype**

Rater 1

|  | **0** | **1** | **2** | **3** | **4** | **Missing** | **Total** |  |
| --- | --- | --- | --- | --- | --- | --- | --- | --- |
| **0** | 856 | 16 | 0 | 6 | 2 | 0 | 880 | [99,4%] |
| **1** | 0 | 0 | 0 | 0 | 0 | 0 | 0 | [0%] |
| **2** | 0 | 0 | 0 | 0 | 0 | 0 | 0 | [0%] |
| **3** | 0 | 0 | 0 | 1 | 0 | 0 | 1 | [0,1%] |
| **4** | 2 | 0 | 0 | 2 | 0 | 0 | 4 | [0,5%] |
| **Missing** | 0 | 0 | 0 | 0 | 0 | 0 | 0 | [0%] |
| **Total** | 858 | 16 | 0 | 9 | 2 | 0 | 885 | [100%] |
| [96,9%] | | [1,8%] | [0%] | [1%] | [0,2%] | [0%] | [100%] |  |

DISTRIBUTION OF SUBJECTS BY RATER AND CATEGORY (0=No change, 1=Appeared, 2=Disappeared; 3=Worsened and 4=Improved) Rater 3

## INTER-RATER RELIABILITY COEFFICIENTS AND ASSOCIATED PRECISION MEASURES

Unweighted Agreement Coefficients

| METHOD | **Coeff.** | **StdErr** | **95% C.I.** | **p-Value** |
| --- | --- | --- | --- | --- |
| **Cohen's Kappa** | 0,12028 | 0,06612987 | -0,01 to 0,25 | 6,928E-02 |
| **Gwet's AC_1_** | 0,96808 | 0,005989556 | 0,956 to 0,98 | 0,000E+00 |
| **Scott's Pi** | 0,11392 | 0,067536936 | -0,019 to 0,246 | 9,199E-02 |
| **Krippendorff's Alpha** | 0,11442 | 0,067536936 | -0,018 to 0,247 | 9,057E-02 |
| **Brenann-Prediger** | 0,96045 | 0,007358851 | 0,946 to 0,975 | 0,000E+00 |
| **Percent Agreement** | 0,96836 | 0,005887081 | 0,957 to 0,98 | 0,000E+00 |

# LANDIS-KOCH INTERPRETATION OF THE AGREEMENT COEFFICIENTS

Benchmarking Unweighted Agreement Coefficients using Cumulative Membership Probabilities

| **Benchmark** | **Interpretation** | **Cohen** | **Gwet** | **Scott's** | **Krippendorff** | **Brennan** | **Percent** |
| --- | --- | --- | --- | --- | --- | --- | --- |
| **Scale** |  | **Kappa** | **AC_1_** | **Pi** | **Alpha** | **Prediger** | **Agreement** |
| 0,8 to 1 | Almost Perfect | 0,00000 | 1,00000 | 0,00000 | 0,00000 | 1,00000 | 1,00000 |
| 0,6 to 0,8 | Substantial | 0,00000 | 1,00000 | 0,00000 | 0,00000 | 1,00000 | 1,00000 |
| 0,4 to 0,6 | Moderate | 0,00001 | 1,00000 | 0,00001 | 0,00001 | 1,00000 | 1,00000 |
| 0,2 to 0,4 | Fair | 0,11400 | 1,00000 | 0,10124 | 0,10256 | 1,00000 | 1,00000 |
| 0 to 0,2 | Slight | 0,96553 | 1,00000 | 0,95418 | 0,95489 | 1,00000 | 1,00000 |
| Less than 0 | Poor | 1,00000 | 1,00000 | 1,00000 | 1,00000 | 1,00000 | 1,00000 |

**Group: Facet degeneration MR14changetype**

Rater 1

|  | **0** | **1** | **2** | **3** | **4** | **Missing** | **Total** |  |
| --- | --- | --- | --- | --- | --- | --- | --- | --- |
| **0** | 529 | 2 | 0 | 0 | 0 | 0 | 531 | [100%] |
| **1** | 0 | 0 | 0 | 0 | 0 | 0 | 0 | [0%] |
| **2** | 0 | 0 | 0 | 0 | 0 | 0 | 0 | [0%] |
| **3** | 0 | 0 | 0 | 0 | 0 | 0 | 0 | [0%] |
| **4** | 0 | 0 | 0 | 0 | 0 | 0 | 0 | [0%] |
| **Missing** | 0 | 0 | 0 | 0 | 0 | 0 | 0 | [0%] |
| **Total** | 529 | 2 | 0 | 0 | 0 | 0 | 531 | [100%] |
| [99,6%] | | [0,4%] | [0%] | [0%] | [0%] | [0%] | [100%] |  |

DISTRIBUTION OF SUBJECTS BY RATER AND CATEGORY (0=No change, 1=Appeared, 2=Disappeared; 3=Worsened and 4=Improved) Rater 3

## INTER-RATER RELIABILITY COEFFICIENTS AND ASSOCIATED PRECISION MEASURES

Unweighted Agreement Coefficients

| METHOD | **Coeff.** | **StdErr** | **95% C.I.** | **p-Value** |
| --- | --- | --- | --- | --- |
| **Cohen's Kappa** | 0,00000 | 3,86983E-17 | 0 to 0 | n/a |
| **Gwet's AC_1_** | 0,99623 | 0,002665792 | 0,991 to 1 | 0,000E+00 |
| **Scott's Pi** | -0,00189 | 0,001335419 | -0,005 to 0,001 | 1,583E-01 |
| **Krippendorff's Alpha** | -0,00094 | 0,001335419 | -0,004 to 0,002 | 4,802E-01 |

| **Brenann-Prediger** | 0,99529 | 0,003325986 | 0,989 to 1 | 0,000E+00 |
| --- | --- | --- | --- | --- |
| **Percent Agreement** | 0,99623 | 0,002660789 | 0,991 to 1 | 0,000E+00 |

# LANDIS-KOCH INTERPRETATION OF THE AGREEMENT COEFFICIENTS

Benchmarking Unweighted Agreement Coefficients using Cumulative Membership Probabilities

| **Benchmark** | **Interpretation** | **Cohen** | **Gwet** | **Scott's** | **Krippendorff** | **Brennan** | **Percent** |
| --- | --- | --- | --- | --- | --- | --- | --- |
| **Scale** |  | **Kappa** | **AC_1_** | **Pi** | **Alpha** | **Prediger** | **Agreement** |
| 0,8 to 1 | Almost Perfect | 0,00000 | 1,00000 | 0,00000 | 0,00000 | 1,00000 | 1,00000 |
| 0,6 to 0,8 | Substantial | 0,00000 | 1,00000 | 0,00000 | 0,00000 | 1,00000 | 1,00000 |
| 0,4 to 0,6 | Moderate | 0,00000 | 1,00000 | 0,00000 | 0,00000 | 1,00000 | 1,00000 |
| 0,2 to 0,4 | Fair | 0,00000 | 1,00000 | 0,00000 | 0,00000 | 1,00000 | 1,00000 |
| 0 to 0,2 | Slight | 0,50000 | 1,00000 | 0,07884 | 0,23996 | 1,00000 | 1,00000 |
| Less than 0 | Poor | 1,00000 | 1,00000 | 1,00000 | 1,00000 | 1,00000 | 1,00000 |

**Group: Overall**

Rater 1

|  | **0** | **1** | **2** | **3** | **4** | **Missing** | **Total** |  |
| --- | --- | --- | --- | --- | --- | --- | --- | --- |
| **0** | 5833 | 93 | 17 | 32 | 3 | 0 | 5978 | [99,3%] |
| **1** | 10 | 5 | 2 | 1 | 0 | 0 | 18 | [0,3%] |
| **2** | 6 | 0 | 1 | 0 | 0 | 0 | 7 | [0,1%] |
| **3** | 3 | 1 | 0 | 1 | 0 | 0 | 5 | [0,1%] |
| **4** | 6 | 0 | 2 | 2 | 0 | 0 | 10 | [0,2%] |
| **Missing** | 0 | 0 | 0 | 0 | 0 | 0 | 0 | [0%] |
| **Total** | 5858 | 99 | 22 | 36 | 3 | 0 | 6018 | [100%] |
| [97,3%] | | [1,6%] | [0,4%] | [0,6%] | [0%] | [0%] | [100%] |  |

DISTRIBUTION OF SUBJECTS BY RATER AND CATEGORY (0=No change, 1=Appeared, 2=Disappeared; 3=Worsened and 4=Improved) Rater 3

| INTER-RATER RELIABILITY COEFFICIENTS AND ASSOCIATED PRECISION MEASURES |
| --- |
| Unweighted Agreement Coefficients |

| METHOD | **Coeff.** | **StdErr** | **95% C.I.** | **p-Value** |
| --- | --- | --- | --- | --- |
| **Cohen's Kappa** | 0,10364 | 0,026763405 | 0,051 to 0,156 | 1,090E-04 |
| **Gwet's AC_1_** | 0,97018 | 0,002218921 | 0,966 to 0,975 | 0,000E+00 |
| **Scott's Pi** | 0,09945 | 0,027132661 | 0,046 to 0,153 | 2,490E-04 |
| **Krippendorff's Alpha** | 0,09953 | 0,027132661 | 0,046 to 0,153 | 2,463E-04 |
| **Brenann-Prediger** | 0,96303 | 0,002730136 | 0,958 to 0,968 | 0,000E+00 |
| **Percent Agreement** | 0,97042 | 0,002184109 | 0,966 to 0,975 | 0,000E+00 |

# LANDIS-KOCH INTERPRETATION OF THE AGREEMENT COEFFICIENTS

Benchmarking Unweighted Agreement Coefficients using Cumulative Membership Probabilities

| **Benchmark** | **Interpretation** | **Cohen** | **Gwet** | **Scott's** | **Krippendorff** | **Brennan** | **Percent** |
| --- | --- | --- | --- | --- | --- | --- | --- |
| **Scale** |  | **Kappa** | **AC_1_** | **Pi** | **Alpha** | **Prediger** | **Agreement** |
| 0,8 to 1 | Almost Perfect | 0,00000 | 1,00000 | 0,00000 | 0,00000 | 1,00000 | 1,00000 |
| 0,6 to 0,8 | Substantial | 0,00000 | 1,00000 | 0,00000 | 0,00000 | 1,00000 | 1,00000 |
| 0,4 to 0,6 | Moderate | 0,00000 | 1,00000 | 0,00000 | 0,00000 | 1,00000 | 1,00000 |
| 0,2 to 0,4 | Fair | 0,00016 | 1,00000 | 0,00011 | 0,00011 | 1,00000 | 1,00000 |
| 0 to 0,2 | Slight | 0,99995 | 1,00000 | 0,99988 | 0,99988 | 1,00000 | 1,00000 |
| Less than 0 | Poor | 1,00000 | 1,00000 | 1,00000 | 1,00000 | 1,00000 | 1,00000 |
