# Supplementary material for: Reliability of reporting differences in degenerative MRI findings of the lumbar spine from the supine to the upright position
Source: Skeletal Radiol. 2022 May 10;51(11):2141–54. doi: 10.1007/s00256-022-04060-2 (PMC9463326; doi:10.1007/s00256-022-04060-2)
Supplement: Supplementary file 4 — Supplementary file4 (DOCX 38 KB) [file 256_2022_4060_MOESM4_ESM.docx]

| **Inter-rater reliability of observed differences comparing** |
| --- |
| **degenerative MRI-findings in the supine and upright positions** |
| **AgreeStat 2015.6.1** |
| MODULE: Two-Rater Chance-Corrected Agreement Coefficients (Time: 16:52:24. Date: 14. juli 2020) |

**Group: MR02 Spondylolisthesis changetype**

Rater 2

|  | **0** | **1** | **2** | **3** | **4** | **Missing** | **Total** |  |
| --- | --- | --- | --- | --- | --- | --- | --- | --- |
| **0** | 528 | 0 | 1 | 1 | 0 | 0 | 530 | [99,8%] |
| **1** | 0 | 0 | 0 | 0 | 0 | 0 | 0 | [0%] |
| **2** | 0 | 0 | 0 | 0 | 0 | 0 | 0 | [0%] |
| **3** | 0 | 0 | 0 | 1 | 0 | 0 | 1 | [0,2%] |
| **4** | 0 | 0 | 0 | 0 | 0 | 0 | 0 | [0%] |
| **Missing** | 0 | 0 | 0 | 0 | 0 | 0 | 0 | [0%] |
| **Total** | 528 | 0 | 1 | 2 | 0 | 0 | 531 | [100%] |
| [99,4%] | | [0%] | [0,2%] | [0,4%] | [0%] | [0%] | [100%] |  |

DISTRIBUTION OF SUBJECTS BY RATER AND CATEGORY (0=No change, 1=Appeared, 2=Disappeared; 3=Worsened and 4=Improved) Rater 3

## INTER-RATER RELIABILITY COEFFICIENTS AND ASSOCIATED PRECISION MEASURES

Unweighted Agreement Coefficients

| METHOD | **Coeff.** | **StdErr** | **95% C.I.** | **p-Value** |
| --- | --- | --- | --- | --- |
| **Cohen's Kappa** | 0,49882 | 0,306377385 | -0,103 to 1 | 1,041E-01 |
| **Gwet's AC_1_** | 0,99623 | 0,002668287 | 0,991 to 1 | 0,000E+00 |
| **Scott's Pi** | 0,49847 | 0,306957305 | -0,105 to 1 | 1,050E-01 |
| **Krippendorff's Alpha** | 0,49894 | 0,306957305 | -0,104 to 1 | 1,047E-01 |
| **Brenann-Prediger** | 0,99529 | 0,003325986 | 0,989 to 1 | 0,000E+00 |
| **Percent Agreement** | 0,99623 | 0,002660789 | 0,991 to 1 | 0,000E+00 |

# LANDIS-KOCH INTERPRETATION OF THE AGREEMENT COEFFICIENTS

Benchmarking Unweighted Agreement Coefficients using Cumulative Membership Probabilities

| **Benchmark** | **Interpretation** | **Cohen** | **Gwet** | **Scott's** | **Krippendorff** | **Brennan** | **Percent** |
| --- | --- | --- | --- | --- | --- | --- | --- |
| **Scale** |  | **Kappa** | **AC_1_** | **Pi** | **Alpha** | **Prediger** | **Agreement** |
| 0,8 to 1 | Almost Perfect | 0,11786 | 1,00000 | 0,11785 | 0,11810 | 1,00000 | 1,00000 |
| 0,6 to 0,8 | Substantial | 0,33683 | 1,00000 | 0,33647 | 0,33697 | 1,00000 | 1,00000 |
| 0,4 to 0,6 | Moderate | 0,60643 | 1,00000 | 0,60564 | 0,60619 | 1,00000 | 1,00000 |
| 0,2 to 0,4 | Fair | 0,82646 | 1,00000 | 0,82564 | 0,82601 | 1,00000 | 1,00000 |
| 0 to 0,2 | Slight | 0,94547 | 1,00000 | 0,94499 | 0,94515 | 1,00000 | 1,00000 |
| Less than 0 | Poor | 1,00000 | 1,00000 | 1,00000 | 1,00000 | 1,00000 | 1,00000 |

**Group: MR05 Scoliosis changetype**

Rater 2

|  | **0** | **1** | **2** | **3** | **4** | **Missing** | **Total** |  |
| --- | --- | --- | --- | --- | --- | --- | --- | --- |
| **0** | 170 | 3 | 0 | 0 | 0 | 0 | 173 | [97,7%] |
| **1** | 3 | 0 | 0 | 0 | 0 | 0 | 3 | [1,7%] |
| **2** | 0 | 0 | 0 | 0 | 0 | 0 | 0 | [0%] |
| **3** | 1 | 0 | 0 | 0 | 0 | 0 | 1 | [0,6%] |
| **4** | 0 | 0 | 0 | 0 | 0 | 0 | 0 | [0%] |
| **Missing** | 0 | 0 | 0 | 0 | 0 | 0 | 0 | [0%] |
| **Total** | 174 | 3 | 0 | 0 | 0 | 0 | 177 | [100%] |
| [98,3%] | | [1,7%] | [0%] | [0%] | [0%] | [0%] | [100%] |  |

DISTRIBUTION OF SUBJECTS BY RATER AND CATEGORY (0=No change, 1=Appeared, 2=Disappeared; 3=Worsened and 4=Improved) Rater 3

## INTER-RATER RELIABILITY COEFFICIENTS AND ASSOCIATED PRECISION MEASURES

Unweighted Agreement Coefficients

| METHOD | **Coeff.** | **StdErr** | **95% C.I.** | **p-Value** |
| --- | --- | --- | --- | --- |
| **Cohen's Kappa** | -0,01724 | 0,007131837 | -0,031 to -0,003 | 1,665E-02 |

| **Gwet's AC_1_** | 0,96006 | 0,014977846 | 0,931 to 0,99 | 6,080E-124 |
| --- | --- | --- | --- | --- |
| **Scott's Pi** | -0,01766 | 0,006951824 | -0,031 to -0,004 | 1,194E-02 |
| **Krippendorff's Alpha** | -0,01478 | 0,006951824 | -0,029 to -0,001 | 3,484E-02 |
| **Brenann-Prediger** | 0,95056 | 0,018363434 | 0,914 to 0,987 | 1,982E-108 |
| **Percent Agreement** | 0,96045 | 0,014690747 | 0,931 to 0,989 | 2,159E-125 |

# LANDIS-KOCH INTERPRETATION OF THE AGREEMENT COEFFICIENTS

Benchmarking Unweighted Agreement Coefficients using Cumulative Membership Probabilities

| **Benchmark** | **Interpretation** | **Cohen** | **Gwet** | **Scott's** | **Krippendorff** | **Brennan** | **Percent** |
| --- | --- | --- | --- | --- | --- | --- | --- |
| **Scale** |  | **Kappa** | **AC_1_** | **Pi** | **Alpha** | **Prediger** | **Agreement** |
| 0,8 to 1 | Almost Perfect | 0,00000 | 1,00000 | 0,00000 | 0,00000 | 1,00000 | 1,00000 |
| 0,6 to 0,8 | Substantial | 0,00000 | 1,00000 | 0,00000 | 0,00000 | 1,00000 | 1,00000 |
| 0,4 to 0,6 | Moderate | 0,00000 | 1,00000 | 0,00000 | 0,00000 | 1,00000 | 1,00000 |
| 0,2 to 0,4 | Fair | 0,00000 | 1,00000 | 0,00000 | 0,00000 | 1,00000 | 1,00000 |
| 0 to 0,2 | Slight | 0,00781 | 1,00000 | 0,00554 | 0,01672 | 1,00000 | 1,00000 |
| Less than 0 | Poor | 1,00000 | 1,00000 | 1,00000 | 1,00000 | 1,00000 | 1,00000 |

**Group: MR06 Annular fissure changetype**

Rater 2

|  | **0** | **1** | **2** | **3** | **4** | **Missing** | **Total** |  |
| --- | --- | --- | --- | --- | --- | --- | --- | --- |
| **0** | 163 | 2 | 5 | 1 | 0 | 0 | 171 | [96,6%] |
| **1** | 1 | 1 | 0 | 0 | 0 | 0 | 2 | [1,1%] |
| **2** | 4 | 0 | 0 | 0 | 0 | 0 | 4 | [2,3%] |
| **3** | 0 | 0 | 0 | 0 | 0 | 0 | 0 | [0%] |
| **4** | 0 | 0 | 0 | 0 | 0 | 0 | 0 | [0%] |
| **Missing** | 0 | 0 | 0 | 0 | 0 | 0 | 0 | [0%] |
| **Total** | 168 | 3 | 5 | 1 | 0 | 0 | 177 | [100%] |
| [94,9%] | | [1,7%] | [2,8%] | [0,6%] | [0%] | [0%] | [100%] |  |

DISTRIBUTION OF SUBJECTS BY RATER AND CATEGORY (0=No change, 1=Appeared, 2=Disappeared; 3=Worsened and 4=Improved) Rater 3

## INTER-RATER RELIABILITY COEFFICIENTS AND ASSOCIATED PRECISION MEASURES

Unweighted Agreement Coefficients

| METHOD | **Coeff.** | **StdErr** | **95% C.I.** | **p-Value** |
| --- | --- | --- | --- | --- |
| **Cohen's Kappa** | 0,10641 | 0,122936164 | -0,136 to 0,349 | 3,879E-01 |
| **Gwet's AC_1_** | 0,92501 | 0,020424834 | 0,885 to 0,965 | 6,321E-99 |
| **Scott's Pi** | 0,10537 | 0,123284823 | -0,138 to 0,349 | 3,939E-01 |
| **Krippendorff's Alpha** | 0,10789 | 0,123284823 | -0,135 to 0,351 | 3,827E-01 |
| **Brenann-Prediger** | 0,90819 | 0,024579555 | 0,86 to 0,957 | 7,538E-85 |
| **Percent Agreement** | 0,92655 | 0,019663644 | 0,888 to 0,965 | 1,001E-101 |

# LANDIS-KOCH INTERPRETATION OF THE AGREEMENT COEFFICIENTS

Benchmarking Unweighted Agreement Coefficients using Cumulative Membership Probabilities

| **Benchmark** | **Interpretation** | **Cohen** | **Gwet** | **Scott's** | **Krippendorff** | **Brennan** | **Percent** |
| --- | --- | --- | --- | --- | --- | --- | --- |
| **Scale** |  | **Kappa** | **AC_1_** | **Pi** | **Alpha** | **Prediger** | **Agreement** |
| 0,8 to 1 | Almost Perfect | 0,00000 | 1,00000 | 0,00000 | 0,00000 | 0,99999 | 1,00000 |
| 0,6 to 0,8 | Substantial | 0,00003 | 1,00000 | 0,00003 | 0,00003 | 1,00000 | 1,00000 |
| 0,4 to 0,6 | Moderate | 0,00847 | 1,00000 | 0,00843 | 0,00891 | 1,00000 | 1,00000 |
| 0,2 to 0,4 | Fair | 0,22324 | 1,00000 | 0,22136 | 0,22750 | 1,00000 | 1,00000 |
| 0 to 0,2 | Slight | 0,80663 | 1,00000 | 0,80363 | 0,80925 | 1,00000 | 1,00000 |
| Less than 0 | Poor | 1,00000 | 1,00000 | 1,00000 | 1,00000 | 1,00000 | 1,00000 |

**Group: MR07 Disc degeneration changetype**

Rater 2

DISTRIBUTION OF SUBJECTS BY RATER AND CATEGORY (0=No change, 1=Appeared, 2=Disappeared; 3=Worsened and 4=Improved) Rater 3

[99,4%]

|  | **0** | **1** | **2** | **3** | **4** | **Missing** | **Total** |
| --- | --- | --- | --- | --- | --- | --- | --- |
| **0** | 174 | 0 | 0 | 2 | 0 | 0 | 176 |
| **1** | 0 | 0 | 0 | 0 | 0 | 0 | 0 |
| **2** | 0 | 0 | 0 | 0 | 0 | 0 | 0 |
| **3** | 1 | 0 | 0 | 0 | 0 | 0 | 1 |

[0%]

[0%]

[0,6%]

| **4** | 0 | 0 | 0 | 0 | 0 | 0 | 0 | [0%] |
| --- | --- | --- | --- | --- | --- | --- | --- | --- |
| **Missing** | 0 | 0 | 0 | 0 | 0 | 0 | 0 | [0%] |
| **Total** | 175 | 0 | 0 | 2 | 0 | 0 | 177 | [100%] |
|  | [98,9%] | [0%] | [0%] | [1,1%] | [0%] | [0%] | [100%] |  |

## INTER-RATER RELIABILITY COEFFICIENTS AND ASSOCIATED PRECISION MEASURES

Unweighted Agreement Coefficients

| METHOD | **Coeff.** | **StdErr** | **95% C.I.** | **p-Value** |
| --- | --- | --- | --- | --- |
| **Cohen's Kappa** | -0,00759 | 0,005392386 | -0,018 to 0,003 | 1,610E-01 |
| **Gwet's AC_1_** | 0,98298 | 0,009811762 | 0,964 to 1 | 3,826E-157 |
| **Scott's Pi** | -0,00855 | 0,004948436 | -0,018 to 0,001 | 8,588E-02 |
| **Krippendorff's Alpha** | -0,00570 | 0,004948436 | -0,015 to 0,004 | 2,511E-01 |
| **Brenann-Prediger** | 0,97881 | 0,012162299 | 0,955 to 1 | 9,212E-141 |
| **Percent Agreement** | 0,98305 | 0,009729839 | 0,964 to 1 | 8,856E-158 |

# LANDIS-KOCH INTERPRETATION OF THE AGREEMENT COEFFICIENTS

Benchmarking Unweighted Agreement Coefficients using Cumulative Membership Probabilities

| **Benchmark** | **Interpretation** | **Cohen** | **Gwet** | **Scott's** | **Krippendorff** | **Brennan** | **Percent** |
| --- | --- | --- | --- | --- | --- | --- | --- |
| **Scale** |  | **Kappa** | **AC_1_** | **Pi** | **Alpha** | **Prediger** | **Agreement** |
| 0,8 to 1 | Almost Perfect | 0,00000 | 1,00000 | 0,00000 | 0,00000 | 1,00000 | 1,00000 |
| 0,6 to 0,8 | Substantial | 0,00000 | 1,00000 | 0,00000 | 0,00000 | 1,00000 | 1,00000 |
| 0,4 to 0,6 | Moderate | 0,00000 | 1,00000 | 0,00000 | 0,00000 | 1,00000 | 1,00000 |
| 0,2 to 0,4 | Fair | 0,00000 | 1,00000 | 0,00000 | 0,00000 | 1,00000 | 1,00000 |
| 0 to 0,2 | Slight | 0,07963 | 1,00000 | 0,04206 | 0,12477 | 1,00000 | 1,00000 |
| Less than 0 | Poor | 1,00000 | 1,00000 | 1,00000 | 1,00000 | 1,00000 | 1,00000 |

**Group: MR08 Disc contour changetype**

| DISTRIBUTION OF SUBJECTS BY RATER AND CATEGORY (0=No change, 1=Appeared, 2=Disappeared; 3=Worsened and 4=Improved) |
| --- |
| Rater 3 |

Rater 2

|  | **0** | **1** | **2** | **3** | **4** | **Missing** | **Total** |  |
| --- | --- | --- | --- | --- | --- | --- | --- | --- |
| **0** | 319 | 4 | 0 | 15 | 1 | 0 | 339 | [95,8%] |
| **1** | 2 | 1 | 0 | 2 | 0 | 0 | 5 | [1,4%] |
| **2** | 1 | 0 | 0 | 0 | 0 | 0 | 1 | [0,3%] |
| **3** | 6 | 0 | 0 | 3 | 0 | 0 | 9 | [2,5%] |
| **4** | 0 | 0 | 0 | 0 | 0 | 0 | 0 | [0%] |
| **Missing** | 0 | 0 | 0 | 0 | 0 | 0 | 0 | [0%] |
| **Total** | 328 | 5 | 0 | 20 | 1 | 0 | 354 | [100%] |
|  | [92,7%] | [1,4%] | [0%] | [5,6%] | [0,3%] | [0%] | [100%] |  |

## INTER-RATER RELIABILITY COEFFICIENTS AND ASSOCIATED PRECISION MEASURES

Unweighted Agreement Coefficients

| METHOD | **Coeff.** | **StdErr** | **95% C.I.** | **p-Value** |
| --- | --- | --- | --- | --- |
| **Cohen's Kappa** | 0,21158 | 0,0837149 | 0,047 to 0,376 | 1,193E-02 |
| **Gwet's AC_1_** | 0,90994 | 0,015804369 | 0,879 to 0,941 | 1,630E-181 |
| **Scott's Pi** | 0,20811 | 0,084615753 | 0,042 to 0,375 | 1,439E-02 |
| **Krippendorff's Alpha** | 0,20923 | 0,084615753 | 0,043 to 0,376 | 1,388E-02 |
| **Brenann-Prediger** | 0,89054 | 0,01880622 | 0,854 to 0,928 | 5,450E-155 |
| **Percent Agreement** | 0,91243 | 0,015044976 | 0,883 to 0,942 | 9,414E-189 |

# LANDIS-KOCH INTERPRETATION OF THE AGREEMENT COEFFICIENTS

Benchmarking Unweighted Agreement Coefficients using Cumulative Membership Probabilities

| **Benchmark** | **Interpretation** | **Cohen** | **Gwet** | **Scott's** | **Krippendorff** | **Brennan** | **Percent** |
| --- | --- | --- | --- | --- | --- | --- | --- |
| **Scale** |  | **Kappa** | **AC_1_** | **Pi** | **Alpha** | **Prediger** | **Agreement** |
| 0,8 to 1 | Almost Perfect | 0,00000 | 1,00000 | 0,00000 | 0,00000 | 1,00000 | 1,00000 |
| 0,6 to 0,8 | Substantial | 0,00000 | 1,00000 | 0,00000 | 0,00000 | 1,00000 | 1,00000 |
| 0,4 to 0,6 | Moderate | 0,01220 | 1,00000 | 0,01167 | 0,01208 | 1,00000 | 1,00000 |
| 0,2 to 0,4 | Fair | 0,55501 | 1,00000 | 0,53818 | 0,54343 | 1,00000 | 1,00000 |
| 0 to 0,2 | Slight | 0,99425 | 1,00000 | 0,99304 | 0,99330 | 1,00000 | 1,00000 |
| Less than 0 | Poor | 1,00000 | 1,00000 | 1,00000 | 1,00000 | 1,00000 | 1,00000 |

**Group: MR09 Nerve kompromise changetype**

Rater 2

|  | **0** | **1** | **2** | **3** | **4** | **Missing** | **Total** |  |
| --- | --- | --- | --- | --- | --- | --- | --- | --- |
| **0** | 161 | 5 | 0 | 2 | 0 | 0 | 168 | [94,9%] |
| **1** | 3 | 1 | 0 | 0 | 0 | 0 | 4 | [2,3%] |
| **2** | 0 | 0 | 0 | 0 | 0 | 0 | 0 | [0%] |
| **3** | 5 | 0 | 0 | 0 | 0 | 0 | 5 | [2,8%] |
| **4** | 0 | 0 | 0 | 0 | 0 | 0 | 0 | [0%] |
| **Missing** | 0 | 0 | 0 | 0 | 0 | 0 | 0 | [0%] |
| **Total** | 169 | 6 | 0 | 2 | 0 | 0 | 177 | [100%] |
| [95,5%] | | [3,4%] | [0%] | [1,1%] | [0%] | [0%] | [100%] |  |

DISTRIBUTION OF SUBJECTS BY RATER AND CATEGORY (0=No change, 1=Appeared, 2=Disappeared; 3=Worsened and 4=Improved) Rater 3

## INTER-RATER RELIABILITY COEFFICIENTS AND ASSOCIATED PRECISION MEASURES

Unweighted Agreement Coefficients

| METHOD | **Coeff.** | **StdErr** | **95% C.I.** | **p-Value** |
| --- | --- | --- | --- | --- |
| **Cohen's Kappa** | 0,08543 | 0,109173613 | -0,13 to 0,301 | 4,350E-01 |
| **Gwet's AC_1_** | 0,91325 | 0,021918471 | 0,87 to 0,957 | 4,295E-93 |
| **Scott's Pi** | 0,08432 | 0,109512325 | -0,132 to 0,3 | 4,423E-01 |
| **Krippendorff's Alpha** | 0,08691 | 0,109512325 | -0,129 to 0,303 | 4,285E-01 |
| **Brenann-Prediger** | 0,89407 | 0,026241191 | 0,842 to 0,946 | 2,084E-79 |
| **Percent Agreement** | 0,91525 | 0,020992953 | 0,874 to 0,957 | 2,965E-96 |

# LANDIS-KOCH INTERPRETATION OF THE AGREEMENT COEFFICIENTS

Benchmarking Unweighted Agreement Coefficients using Cumulative Membership Probabilities

| **Benchmark** | **Interpretation** | **Cohen** | **Gwet** | **Scott's** | **Krippendorff** | **Brennan** | **Percent** |
| --- | --- | --- | --- | --- | --- | --- | --- |
| **Scale** |  | **Kappa** | **AC_1_** | **Pi** | **Alpha** | **Prediger** | **Agreement** |
| 0,8 to 1 | Almost Perfect | 0,00000 | 1,00000 | 0,00000 | 0,00000 | 0,99983 | 1,00000 |
| 0,6 to 0,8 | Substantial | 0,00000 | 1,00000 | 0,00000 | 0,00000 | 1,00000 | 1,00000 |

| 0,4 to 0,6 | Moderate | 0,00198 | 1,00000 | 0,00197 | 0,00213 | 1,00000 | 1,00000 |
| --- | --- | --- | --- | --- | --- | --- | --- |
| 0,2 to 0,4 | Fair | 0,14699 | 1,00000 | 0,14542 | 0,15088 | 1,00000 | 1,00000 |
| 0 to 0,2 | Slight | 0,78304 | 1,00000 | 0,77935 | 0,78629 | 1,00000 | 1,00000 |
| Less than 0 | Poor | 1,00000 | 1,00000 | 1,00000 | 1,00000 | 1,00000 | 1,00000 |

**Group: MR12 Spinal stenosis changetype**

Rater 2

|  | **0** | **1** | **2** | **3** | **4** | **Missing** | **Total** |  |
| --- | --- | --- | --- | --- | --- | --- | --- | --- |
| **0** | 834 | 12 | 0 | 6 | 1 | 0 | 853 | [96,4%] |
| **1** | 9 | 2 | 0 | 1 | 0 | 0 | 12 | [1,4%] |
| **2** | 0 | 0 | 0 | 0 | 0 | 0 | 0 | [0%] |
| **3** | 14 | 2 | 0 | 2 | 1 | 0 | 19 | [2,1%] |
| **4** | 1 | 0 | 0 | 0 | 0 | 0 | 1 | [0,1%] |
| **Missing** | 0 | 0 | 0 | 0 | 0 | 0 | 0 | [0%] |
| **Total** | 858 | 16 | 0 | 9 | 2 | 0 | 885 | [100%] |
| [96,9%] | | [1,8%] | [0%] | [1%] | [0,2%] | [0%] | [100%] |  |

DISTRIBUTION OF SUBJECTS BY RATER AND CATEGORY (0=No change, 1=Appeared, 2=Disappeared; 3=Worsened and 4=Improved) Rater 3

## INTER-RATER RELIABILITY COEFFICIENTS AND ASSOCIATED PRECISION MEASURES

Unweighted Agreement Coefficients

| METHOD | **Coeff.** | **StdErr** | **95% C.I.** | **p-Value** |
| --- | --- | --- | --- | --- |
| **Cohen's Kappa** | 0,18419 | 0,062615016 | 0,061 to 0,307 | 3,351E-03 |
| **Gwet's AC_1_** | 0,94601 | 0,00777314 | 0,931 to 0,961 | 0,000E+00 |
| **Scott's Pi** | 0,18362 | 0,062701999 | 0,061 to 0,307 | 3,494E-03 |
| **Krippendorff's Alpha** | 0,18408 | 0,062701999 | 0,061 to 0,307 | 3,413E-03 |
| **Brenann-Prediger** | 0,93362 | 0,009427826 | 0,915 to 0,952 | 0,000E+00 |
| **Percent Agreement** | 0,94689 | 0,007542261 | 0,932 to 0,962 | 0,000E+00 |

# LANDIS-KOCH INTERPRETATION OF THE AGREEMENT COEFFICIENTS

Benchmarking Unweighted Agreement Coefficients using Cumulative Membership Probabilities

| **Benchmark** | **Interpretation** | **Cohen** | **Gwet** | **Scott's** | **Krippendorff** | **Brennan** | **Percent** |
| --- | --- | --- | --- | --- | --- | --- | --- |
| **Scale** |  | **Kappa** | **AC_1_** | **Pi** | **Alpha** | **Prediger** | **Agreement** |
| 0,8 to 1 | Almost Perfect | 0,00000 | 1,00000 | 0,00000 | 0,00000 | 1,00000 | 1,00000 |
| 0,6 to 0,8 | Substantial | 0,00000 | 1,00000 | 0,00000 | 0,00000 | 1,00000 | 1,00000 |
| 0,4 to 0,6 | Moderate | 0,00028 | 1,00000 | 0,00028 | 0,00029 | 1,00000 | 1,00000 |
| 0,2 to 0,4 | Fair | 0,40032 | 1,00000 | 0,39695 | 0,39979 | 1,00000 | 1,00000 |
| 0 to 0,2 | Slight | 0,99837 | 1,00000 | 0,99830 | 0,99834 | 1,00000 | 1,00000 |
| Less than 0 | Poor | 1,00000 | 1,00000 | 1,00000 | 1,00000 | 1,00000 | 1,00000 |

**Group: MR14changetyp**

Rater 2

|  | **0** | **1** | **2** | **3** | **4** | **Missing** | **Total** |  |
| --- | --- | --- | --- | --- | --- | --- | --- | --- |
| **0** | 526 | 2 | 0 | 0 | 0 | 0 | 528 | [99,4%] |
| **1** | 3 | 0 | 0 | 0 | 0 | 0 | 3 | [0,6%] |
| **2** | 0 | 0 | 0 | 0 | 0 | 0 | 0 | [0%] |
| **3** | 0 | 0 | 0 | 0 | 0 | 0 | 0 | [0%] |
| **4** | 0 | 0 | 0 | 0 | 0 | 0 | 0 | [0%] |
| **Missing** | 0 | 0 | 0 | 0 | 0 | 0 | 0 | [0%] |
| **Total** | 529 | 2 | 0 | 0 | 0 | 0 | 531 | [100%] |
| [99,6%] | | [0,4%] | [0%] | [0%] | [0%] | [0%] | [100%] |  |

DISTRIBUTION OF SUBJECTS BY RATER AND CATEGORY (0=No change, 1=Appeared, 2=Disappeared; 3=Worsened and 4=Improved) Rater 3

## INTER-RATER RELIABILITY COEFFICIENTS AND ASSOCIATED PRECISION MEASURES

Unweighted Agreement Coefficients

| METHOD | **Coeff.** | **StdErr** | **95% C.I.** | **p-Value** |
| --- | --- | --- | --- | --- |
| **Cohen's Kappa** | -0,00454 | 0,002196289 | -0,009 to 0 | 3,919E-02 |
| **Gwet's AC_1_** | 0,99056 | 0,004214811 | 0,982 to 0,999 | 0,000E+00 |
| **Scott's Pi** | -0,00473 | 0,002117456 | -0,009 to -0,001 | 2,590E-02 |
| **Krippendorff's Alpha** | -0,00378 | 0,002117456 | -0,008 to 0 | 7,448E-02 |

| **Brenann-Prediger** | 0,98823 | 0,005243912 | 0,978 to 0,999 | 0,000E+00 |
| --- | --- | --- | --- | --- |
| **Percent Agreement** | 0,99058 | 0,00419513 | 0,982 to 0,999 | 0,000E+00 |

# LANDIS-KOCH INTERPRETATION OF THE AGREEMENT COEFFICIENTS

Benchmarking Unweighted Agreement Coefficients using Cumulative Membership Probabilities

| **Benchmark** | **Interpretation** | **Cohen** | **Gwet** | **Scott's** | **Krippendorff** | **Brennan** | **Percent** |
| --- | --- | --- | --- | --- | --- | --- | --- |
| **Scale** |  | **Kappa** | **AC_1_** | **Pi** | **Alpha** | **Prediger** | **Agreement** |
| 0,8 to 1 | Almost Perfect | 0,00000 | 1,00000 | 0,00000 | 0,00000 | 1,00000 | 1,00000 |
| 0,6 to 0,8 | Substantial | 0,00000 | 1,00000 | 0,00000 | 0,00000 | 1,00000 | 1,00000 |
| 0,4 to 0,6 | Moderate | 0,00000 | 1,00000 | 0,00000 | 0,00000 | 1,00000 | 1,00000 |
| 0,2 to 0,4 | Fair | 0,00000 | 1,00000 | 0,00000 | 0,00000 | 1,00000 | 1,00000 |
| 0 to 0,2 | Slight | 0,01935 | 1,00000 | 0,01274 | 0,03695 | 1,00000 | 1,00000 |
| Less than 0 | Poor | 1,00000 | 1,00000 | 1,00000 | 1,00000 | 1,00000 | 1,00000 |

**Group: Overall**

Rater 2

|  | **0** | **1** | **2** | **3** | **4** | **Missing** | **Total** |  |
| --- | --- | --- | --- | --- | --- | --- | --- | --- |
| **0** | 5747 | 77 | 20 | 27 | 2 | 0 | 5873 | [97,6%] |
| **1** | 45 | 11 | 1 | 3 | 0 | 0 | 60 | [1%] |
| **2** | 38 | 9 | 1 | 0 | 0 | 0 | 48 | [0,8%] |
| **3** | 27 | 2 | 0 | 6 | 1 | 0 | 36 | [0,6%] |
| **4** | 1 | 0 | 0 | 0 | 0 | 0 | 1 | [0%] |
| **Missing** | 0 | 0 | 0 | 0 | 0 | 0 | 0 | [0%] |
| **Total** | 5858 | 99 | 22 | 36 | 3 | 0 | 6018 | [100%] |
| [97,3%] | | [1,6%] | [0,4%] | [0,6%] | [0%] | [0%] | [100%] |  |

DISTRIBUTION OF SUBJECTS BY RATER AND CATEGORY (0=No change, 1=Appeared, 2=Disappeared; 3=Worsened and 4=Improved) Rater 3

| INTER-RATER RELIABILITY COEFFICIENTS AND ASSOCIATED PRECISION MEASURES |
| --- |
| Unweighted Agreement Coefficients |

| METHOD | **Coeff.** | **StdErr** | **95% C.I.** | **p-Value** |
| --- | --- | --- | --- | --- |
| **Cohen's Kappa** | 0,15601 | 0,026307682 | 0,104 to 0,208 | 3,194E-09 |
| **Gwet's AC_1_** | 0,95743 | 0,00264823 | 0,952 to 0,963 | 0,000E+00 |
| **Scott's Pi** | 0,15573 | 0,026321905 | 0,104 to 0,207 | 3,476E-09 |
| **Krippendorff's Alpha** | 0,15580 | 0,026321905 | 0,104 to 0,207 | 3,420E-09 |
| **Brenann-Prediger** | 0,94745 | 0,003233909 | 0,941 to 0,954 | 0,000E+00 |
| **Percent Agreement** | 0,95796 | 0,002587127 | 0,953 to 0,963 | 0,000E+00 |

# LANDIS-KOCH INTERPRETATION OF THE AGREEMENT COEFFICIENTS

Benchmarking Unweighted Agreement Coefficients using Cumulative Membership Probabilities

| **Benchmark** | **Interpretation** | **Cohen** | **Gwet** | **Scott's** | **Krippendorff** | **Brennan** | **Percent** |
| --- | --- | --- | --- | --- | --- | --- | --- |
| **Scale** |  | **Kappa** | **AC_1_** | **Pi** | **Alpha** | **Prediger** | **Agreement** |
| 0,8 to 1 | Almost Perfect | 0,00000 | 1,00000 | 0,00000 | 0,00000 | 1,00000 | 1,00000 |
| 0,6 to 0,8 | Substantial | 0,00000 | 1,00000 | 0,00000 | 0,00000 | 1,00000 | 1,00000 |
| 0,4 to 0,6 | Moderate | 0,00000 | 1,00000 | 0,00000 | 0,00000 | 1,00000 | 1,00000 |
| 0,2 to 0,4 | Fair | 0,04725 | 1,00000 | 0,04628 | 0,04654 | 1,00000 | 1,00000 |
| 0 to 0,2 | Slight | 1,00000 | 1,00000 | 1,00000 | 1,00000 | 1,00000 | 1,00000 |
| Less than 0 | Poor | 1,00000 | 1,00000 | 1,00000 | 1,00000 | 1,00000 | 1,00000 |
